# Statistical methods for analyzing change over time of continuous outcomes in observational health studies: a scoping review of suggested approaches

**DOI:** 10.1186/s13643-026-03361-0

**Published:** 2026-10-17

**Authors:** Martina Bouka, Eirini Pagkalidou, Solange Gonzalez Chiappe, Tracy Bonsu Osei, Ulrike Grittner, Katharina Nimptsch, Tobias Pischon

**Affiliations:** 1 Molecular Epidemiology Research Group, Max Delbrück Center for Molecular Medicine in the Helmholtz Association (MDC), Berlin, Germany; 2 Institute of Public Health, Charité – University Medical Center Berlin, corporate member of Freie Universität Berlin and Humboldt-Universität zu Berlin, Berlin, Germany; 3 Department of Hygiene and Epidemiology, School of Medicine, University of Ioannina, Ioannina, Greece; 4 Institute of Biometry and Clinical Epidemiology, Charité – University Medical Center Berlin, corporate member of Freie Universität Berlin and Humboldt-Universität zu Berlin, Berlin, Germany; 5 Biobank Technology Platform, Max Delbrück Center for Molecular Medicine in the Helmholtz Association (MDC), Berlin, Germany; 6 Charité – University Medical Center Berlin, corporate member of Freie Universität Berlin and Humboldt-Universität zu Berlin, Berlin, Germany

**Keywords:** Scoping review, Observational longitudinal studies, Within-person change, Regression to the mean, Measurement error, Baseline adjustment

## Abstract

**Background:**

Analyses of within-person change in continuous outcomes are widely used in longitudinal observational health research. However, modeling change presents challenges, particularly regarding questions about when adjustment for baseline outcome values is indicated and how to deal with regression to the mean (RTM) and measurement error (ME). Despite extensive methodological debate, it remains unclear how these issues are addressed in applied studies and what analytical strategies have been proposed to mitigate related biases.

**Methods:**

We conducted a scoping review of methodological studies proposing strategies to address bias when analyzing within-person change in continuous outcomes and of applied observational studies to examine analytical practice following PRISMA guidelines and PROSPERO registration (CRD420251024543). MEDLINE and EMBASE were searched from January 2000 to April 2025. Methodological studies addressed RTM, ME, or related modeling challenges while applied studies examined longitudinal changes in continuous metabolic syndrome (MetS) indicators as well as body weight outcomes. Findings were synthesized narratively and as a decision framework to help researchers select analytical strategies according to the bias of interest.

**Results:**

We included 66 methodological and 102 applied studies. Methodological studies proposed a range of approaches to address bias related to RTM and ME, including analytical RTM correction methods, model-based longitudinal approaches, alternative estimators for baseline-change associations, causal and estimand-based frameworks, calibration methods, latent-variable models, and flexible ME models. Common recommendations were aligning analyses with the research question, accounting for measurement processes, and using repeated measurements where possible. In contrast, applied studies showed heterogeneity in analytical practice. Forty-nine percent used models adjusting for baseline outcome values, 44% used models without baseline adjustment, and 7% reported both. Baseline adjustment was more common in studies examining body weight compared to MetS indicators. Justification for modeling choices, use of causal tools, and addressing related biases was uncommon.

**Conclusions:**

The methodological literature provides a diverse set of approaches addressing RTM and ME in analyses of within-person change, but many of these considerations are not routinely reflected in applied observational research. By synthesizing methodological guidance and comparing it with current analytical practice, this review provides a structured framework to support more transparent and methodologically informed analyses of longitudinal change.

**Supplementary Information:**

The online version contains supplementary material available at https://doi.org/10.1186/s13643-026-03361-0.

## Background

Longitudinal observational data are widely used in health research to investigate how exposures and risk factors relate to changes in health outcomes over time. Quantitative health parameters are frequently measured repeatedly as outcomes in cohort and other observational designs, such as indicators of metabolic dysfunction and the metabolic syndrome (MetS), including body weight, blood pressure (BP), lipid profiles, and glycemic markers. These repeatedly measured outcomes provide opportunities to examine within-person change and its determinants. Large population-based cohort studies, such as the German National Cohort (NAKO) [1], illustrate the growing availability of such longitudinal data in modern epidemiological research. These analyses are central to understanding health-to-disease transition, disease progression, treatment effectiveness in real-world settings, and population health dynamics. However, the statistical modeling of change in continuous outcomes presents important methodological challenges [2, 3], particularly in non-randomized contexts where confounding, measurement error (ME), and model specification can substantially influence results [4].

Among the statistical approaches commonly used to model within-person change in continuous outcomes over time are change-score models, in which change is calculated as the difference between the follow-up and baseline measurement and then analyzed as the outcome variable. In some instances, researchers additionally adjust for the baseline value of the outcome, treating it as a potential confounder of the association between exposure and subsequent change. However, adjusting for baseline values introduces important methodological challenges and has been the subject of longstanding debate [5, 6]. These considerations differ from randomized trials, where randomization ensures comparability of treatment groups in expectation, such that both baseline-adjusted and change-score analyses are generally valid for estimating treatment effects, although baseline adjustment may provide greater statistical efficiency [7–9].

One key concern in longitudinal observational research is mathematical coupling [10], which arises because the baseline measurement is a component of the change variable itself. Another concern is regression to the mean (RTM). This phenomenon occurs when individuals with extreme baseline values naturally have follow-up measurements closer to the population average due to random variation rather than a true underlying change [11, 12].

These issues are closely linked to ME in baseline assessments. When the initial value of an outcome is measured with error, part of the apparent change observed at follow-up may reflect random variation rather than true within-person change, thereby contributing to RTM. In this sense, the observed association between baseline level and subsequent change can arise largely from ME in the initial measurement [6]. Consequently, the problem of RTM in analyses of change is often viewed as, at least in part, a manifestation of ME. This perspective helps explain why statistical approaches designed to account for or correct ME in baseline values are frequently proposed as potential solutions to mitigate bias when examining the relationship between initial values and change over time [13–15].

In response to these concerns, methodological research has proposed a range of strategies to address bias in the analysis of change, including approaches to correct for ME, alternative modeling frameworks, and the use of causal inference tools such as directed acyclic graphs (DAGs) to guide covariate selection and clarify analytical assumptions [16]. Some recent work suggests that when the exposure clearly precedes the baseline measurement of the outcome, unadjusted change-score models may provide more appropriate estimates than models that condition on baseline values [7, 16]. Nevertheless, the question of whether baseline adjustment is warranted is debated and often context dependent.

Despite these methodological developments and ongoing discussions, there is still no clear consensus regarding the most appropriate statistical approach for analyzing within-person change in observational health research. Moreover, it remains unclear how different modeling strategies are implemented in applied studies, how consistently key methodological challenges are addressed, whether analytical choices are adequately justified and transparently reported, and which methods are used in practice to mitigate the related biases. This gap between methodological guidance and analytical practice makes it difficult to interpret findings across studies and to identify best practices for modeling change in continuous outcomes.

To address this, the present scoping review first examines the methodological literature on the analysis of within-person change in continuous outcomes in observational research. Specifically, we synthesize methodological studies that evaluate the implications of baseline adjustment in change-score analyses or describe the sources of bias that may arise from RTM, and ME, and summarize the analytical approaches proposed to mitigate these challenges. We then examine how within-person change is modeled in practice by systematically reviewing observational studies that investigated indicators of body weight and MetS as outcomes as an example. In addition to documenting the statistical approaches used and their frequency in the applied literature, we assess whether and how key methodological challenges are addressed, discussed, and justified in published studies. By integrating evidence from both methodological and applied research, this review aims to inform more transparent and robust analyses of within-person change in future observational health studies.

## Methods

This scoping review was conducted in accordance with the published protocol that was registered in the International Prospective Register of Systematic Reviews (PROSPERO; identifier CRD420251024543) and is reported following the Preferred Reporting Items for Systematic Review and Meta-Analysis extension for scoping reviews (PRISMA-ScR) checklist [17].

### Search strategy and literature screening

We conducted electronic searches in MEDLINE and EMBASE from 01/01/2000, up to 15/04/2025. The year 2000 was chosen in order to focus on methodological approaches and analytical practices used in more recent longitudinal observational health research. The search strategy was developed based on the main concepts of the review and was iteratively refined to include relevant terminology. Reference lists of relevant reviews were also screened to identify additional potentially eligible studies. Details of the search strategy are provided in Additional file 1. Titles and abstracts of all retrieved records were independently screened for eligibility by two reviewers (MB, EP) using the CAMARADES Preclinical Systematic Review & Meta-analysis Facility (SyRF) [18]. Prior to screening, duplicate records were identified and removed using EndNote (Clarivate) [19]. Full-text assessment was conducted for studies considered eligible. Disagreements at any stage were resolved by a third reviewer (KN).

### Study selection

This scoping review comprised two complementary components: (1) a review of methodological studies examining statistical strategies to address bias in modeling change and (2) a review of applied studies that analyze within-person change in continuous indicators of body weight and MetS in observational longitudinal health studies.

Eligible studies were published peer-reviewed research articles. The methodological component included studies addressing modeling strategies or bias mitigation approaches for change in any continuous outcome, irrespective of health domain. Methodological studies were eligible if they discussed statistical methods, simulations, or conceptual frameworks relevant to modeling change in continuous outcomes and addressed methodological challenges such as RTM and ME. For the applied studies, we selected as example studies that examined longitudinal changes in continuous indicators related to MetS, including body weight and the following MetS indicators: high-density lipoprotein cholesterol (HDL-C), hemoglobin A1c (HbA1c), triglycerides (TG), waist circumference (WC), and blood pressure (BP). We selected this example to ensure feasibility and limit heterogeneity in the assessment of analytical practice, and because these outcomes are commonly assessed repeatedly in longitudinal observational health research and are relevant to methodological issues concerning within-person change. Applied studies were eligible if they used observational longitudinal data to model change over time, evaluated change in at least one of the specified continuous outcomes, and included repeated measurements of the outcome. The following exclusion criteria applied to both review components: articles not published in English; studies not involving human participants; randomized controlled trials and purely cross-sectional studies; studies without repeated outcome measurements; studies analyzing only categorical or binary outcomes; studies not evaluating change; and editorials, study protocols, dissertations, and conference abstracts. For the applied component only, reviews, systematic reviews, and meta-analyses were excluded, although their reference lists were screened to identify potentially eligible primary studies. If a single article contained both an applied empirical analysis and a relevant methodological discussion, it was included in both components of the review.

### Data extraction

Data extraction was performed by two researchers (MB, EP), with discrepancies resolved through consultation with a third researcher (KN). Data were extracted using a predefined standardized form. Data extraction was conducted separately for the methodological and applied components. For methodological studies, we extracted study characteristics, the biases addressed (RTM, ME, or others), the bias-mitigation techniques proposed or evaluated, use of causal tools, and key recommendations and limitations. For applied studies, we extracted study characteristics, exposure(s) and outcome(s), timing of measurements, and details of the statistical approach used to model change, including model type, use of baseline covariates, justification for the chosen method, and use of DAGs or other causal frameworks. We also recorded how analyses were framed and clarity of interpretation. Extracted data were stored in a structured format and checked for accuracy prior to synthesis.

### Data synthesis

We conducted a narrative synthesis to summarize and interpret findings separately for the methodological and applied component. Methodological studies were grouped according to the primary bias addressed (RTM, ME, or both). Within these groups studies were classified according to the dominant source of evidence used to support their conclusions. Studies proposing new methods or providing theoretical or conceptual discussions were categorized as methodological or conceptual studies, those relying primarily on simulated data to evaluate methodological performance were classified as simulation studies, and studies applying existing methods to real datasets without proposing new methodology were classified as applied examples. Within each bias category, we summarized study type and grouped the proposed bias-mitigation strategies into conceptual classes based on similarities in their underlying methodological approach and the way in which they addressed the relevant source of bias. These classes were developed during the narrative synthesis based on the approaches identified in the included studies rather than being predefined. We then reported the frequency and proportion of studies within each class. Common strengths and limitations of proposed approaches were synthesized and summarized in tabular form alongside representative examples. We also report the proportion of studies that included applications to real data. Finally, we aimed to use the synthesized findings to develop structured decision trees that map methodological approaches for addressing RTM- and ME-related challenges. For applied studies, we first grouped studies according to whether the statistical model adjusted for baseline outcome values. Studies presenting both baseline-adjusted and non-adjusted models were included in both groups, and we described the frequency of such studies, whether the use of multiple models was justified, and whether results differed across modeling approaches. Within each group, we summarized study design, number of time points, and distribution of outcomes, including proportions of studies examining body weight, BMI, HbA1c, TG, HDL-C, WC, or BP. We examined whether baseline adjustment varied by outcome type and described the statistical models used to analyze change. We summarized whether authors justified their modeling choices, whether analyses were framed as descriptive, explanatory/causal, or predictive, and whether causal tools such as DAGs were used. The main analytical aim of each study was classified based on the stated study objective, the analysis performed, and the measures reported, rather than on the terminology used by the authors alone. Studies were classified as predictive when their main aim was to predict an outcome and predictive performance was evaluated using appropriate measures; the use of terms such as “predictor” or “predicting” without corresponding predictive analyses was not considered sufficient for classification as predictive. We also examined if studies addressed ME and assessed consistency between stated research questions, analytical approach, and interpretation of findings.

## Results

**Fig. 1:**
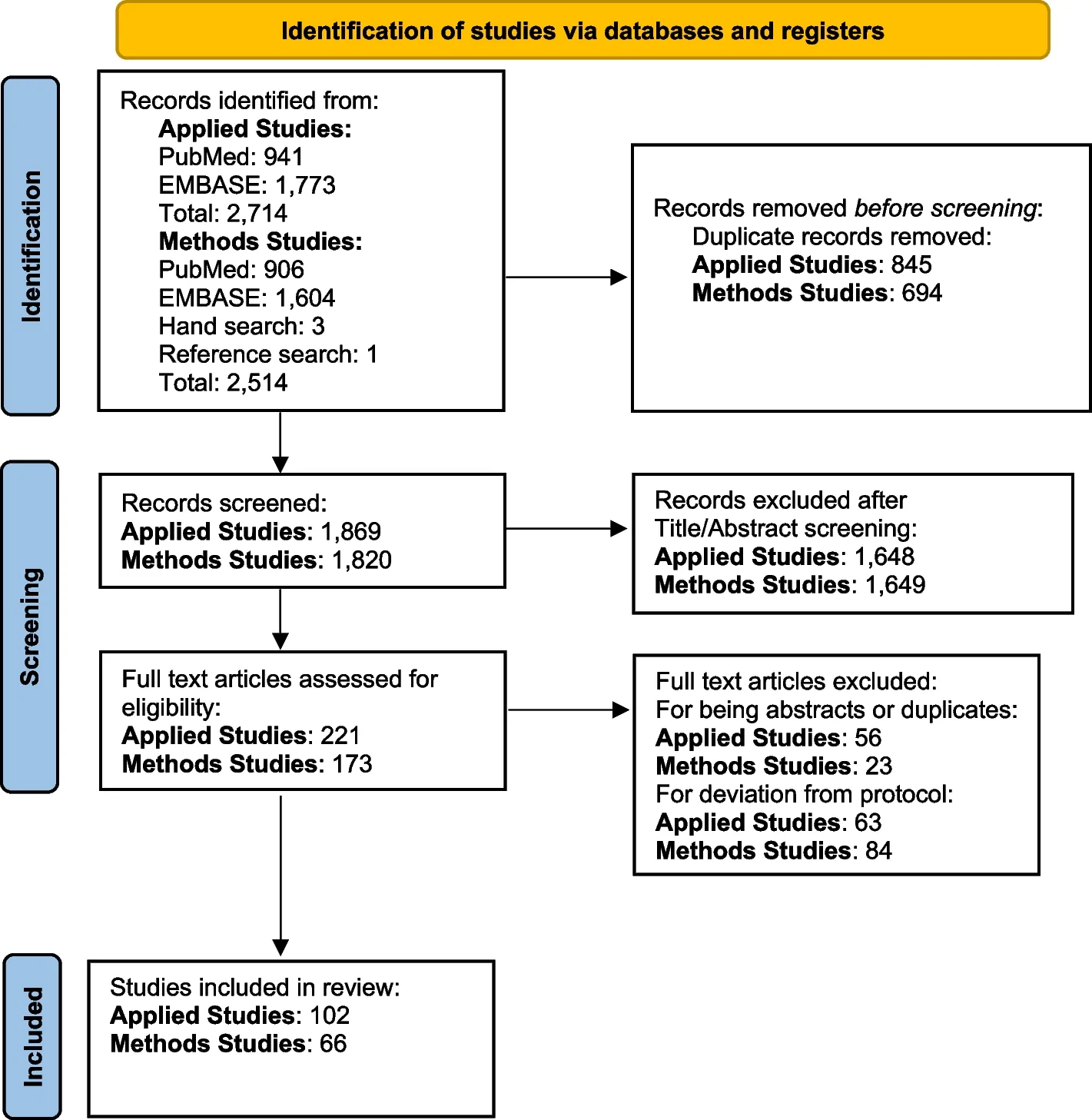
The PRISMA flow diagram

For the methodological component, 2514 records were initially identified, and 66 studies [2, 5–7, 16, 20–80] were ultimately included. For the applied component, 2714 records were initially identified, of which 102 publications [81–182] met the inclusion criteria and were included in the final synthesis (Fig. 1). A detailed list of excluded studies and the corresponding reasons for exclusion is provided in Additional file 2: Sheet S1.

### Methods studies

**Fig. 2:**
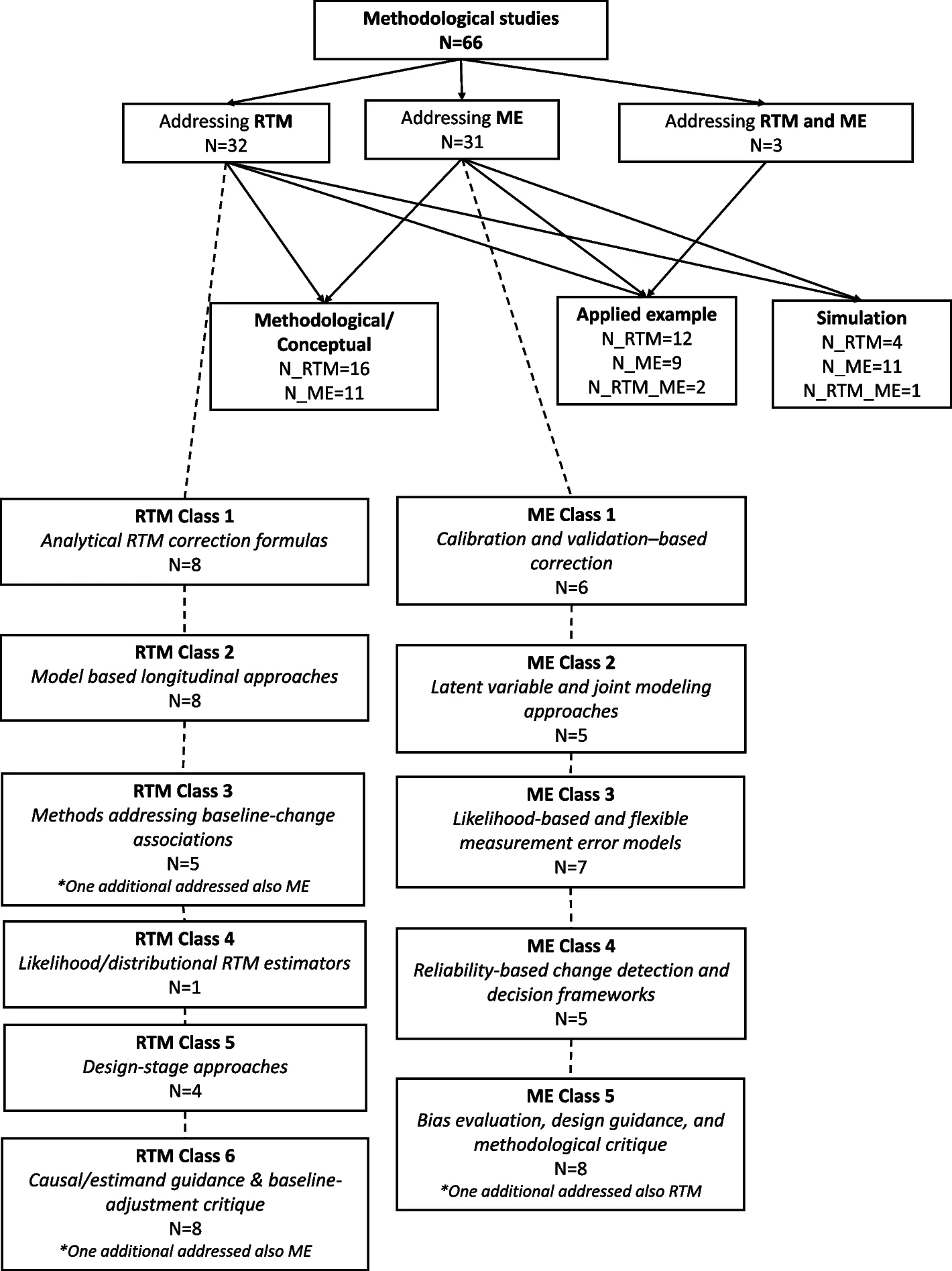
Classification of methodological studies included in the systematic scoping review Legend: Studies were first grouped according to the methodological issue addressed: regression to the mean (RTM), measurement error (ME), or both. They were then classified according to the main source of evidence as methodological/conceptual studies, simulation studies, or applied examples. Approaches identified for addressing RTM and ME were further grouped into six RTM classes and five ME classes based on similarities in their underlying methodological approach. Studies could contribute to more than one category where applicable; therefore, counts across categories are not mutually exclusive. Detailed descriptions of each methodological class, including the studies, strengths, limitations, and recommendations, are provided in Additional file 1: Tables S1 and S2

The methodological studies are summarized in Fig. 2 and Additional file 2: Sheet S2.

### Studies addressing RTM

A total of 32 methodological studies addressed RTM [2, 5–7, 16, 23, 26, 28, 32, 34–37, 41, 43, 49–53, 55, 60, 64, 66–69, 71–74, 77]. Among these, four studies [6, 43, 68, 72] also discussed additional sources of bias, including correlations with residuals, mathematical coupling, and selective attrition. Based on the dominant source of evidence supporting their conclusions, 16 studies were classified as methodological or conceptual [2, 5–7, 16, 23, 28, 35, 49, 51, 52, 60, 66, 68, 72, 77], 12 as applied examples using real data [26, 32, 34, 36, 41, 50, 55, 64, 67, 69, 71, 74], and 4 as simulation studies [37, 43, 53, 73]. Most studies included some form of real-data illustration, with 25 of the 32 studies incorporating empirical applications [2, 3, 23, 26, 28, 32, 34–37, 41, 49–53, 55, 60, 64, 66, 67, 69, 71, 73, 74], while seven studies relied exclusively on methodological exposition or simulation [5–7, 16, 43, 72, 77].

**Fig. 3:**
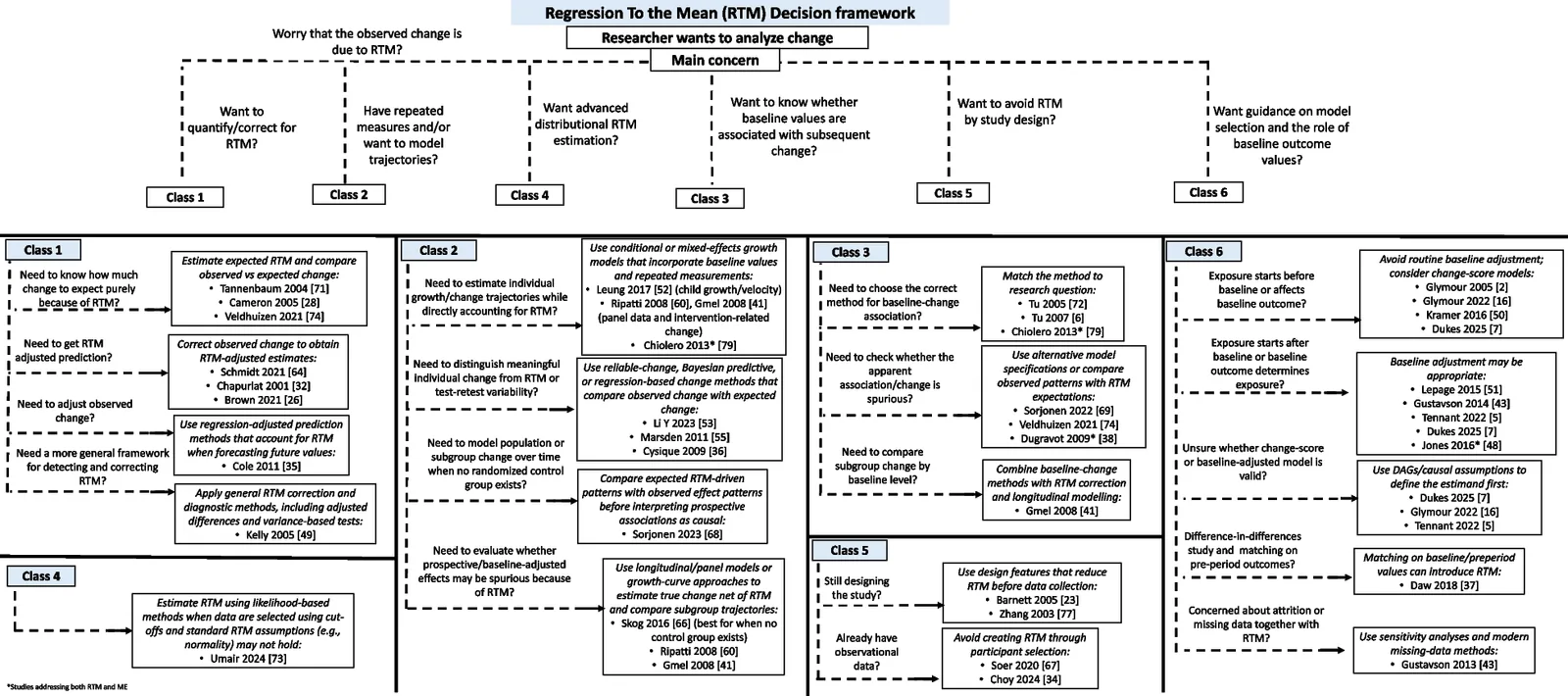
Decision framework for navigating methodological approaches to address regression to mean (RTM) in within-person change analysis Legend: Suggested classification of RTM studies: class 1: analytical RTM correction formulas; class 2: model-based longitudinal approaches; class 3: methods addressing baseline-change associations; class 4: likelihood/distributional RTM estimators; class 5: design-stage approaches; class 6: causal/estimand guidance and baseline-adjustment critique

Across studies, proposed strategies to address RTM clustered into six broad methodological classes (Fig. 3, Additional file 1: Table S1): (1) analytical RTM correction formulas; (2) model-based longitudinal approaches; (3) methods assessing baseline-change associations; (4) likelihood/distributional RTM estimators; (5) design-stage approaches; and (6) causal/estimand guidance and baseline-adjustment critique.

#### Analytical RTM correction formulas

Several studies (*n* = 8) proposed analytical corrections for expected RTM [26, 28, 32, 35, 49, 64, 71, 74]. Specifically, Schmidt et al. [64] and Tannenbaum et al. [71] used algebraically equivalent formulas in which the observed value is shrunk toward the population mean according to the baseline-follow-up correlation. Kelly and Price [49] described the same underlying correction principle. Cameron et al. [28] extended this framework to one which allows different standard deviations at different time points and two other articles [26, 35] used comparable approaches. While the earlier formulas predict an individual’s expected follow-up score given their own baseline value, Veldhuizen et al. [74] estimated the expected RTM shift at the subgroup level following selection on extreme baseline values. Finally, Chapurlat et al. [32] proposed an inverse-variance weighted estimator of an individual’s underlying true baseline value, shrinking observed measurements toward the population mean rather than predicting follow-up values.

#### Model-based longitudinal approaches

A second group of studies (*n* = 8) proposed model-based longitudinal approaches including mixed-effects models, conditional models, and growth-curve frameworks [36, 41, 52, 53, 55, 60, 66, 68]. Rather than applying analytical corrections for expected RTM, these approaches account for RTM within the modeling framework itself by explicitly incorporating baseline values and repeated measurements into the estimation of change. By modeling individual trajectories over time and using information from multiple observations, these methods aim to distinguish true longitudinal change from apparent change attributable to RTM while allowing the effects of exposures, interventions, and covariates to be examined simultaneously.

#### Methods assessing baseline-change associations

Several studies (*n* = 5) focused on methodological approaches for evaluating the relationship between baseline values and subsequent change in the presence of RTM and mathematical coupling [6, 41, 69, 72, 74]. These studies examined methods such as Oldham’s approach, Blomqvist’s correction, correlation-based analyses, and alternative modeling frameworks that seek to distinguish true baseline-change associations from artifacts arising from the statistical dependence between baseline values and change scores.

#### Likelihood-based and distributional estimators

One study proposed likelihood-based estimation of RTM by modeling it explicitly within a statistical distribution. The method uses maximum likelihood estimation to quantify the RTM effect directly from the data and separate it from the observed treatment effect [73]. Unlike analytical correction formulas, which estimate expected RTM using reliability or correlation measures, this approach estimates RTM as part of the model-fitting process.

#### Design-stage approaches

Some studies (*n* = 4) emphasized design-based strategies to reduce RTM, including the use of control groups, repeated baseline measurements, and alternative baseline assessments not used for participant selection [23, 34, 67, 77].

#### Causal and estimand-based guidance

Finally, a group of studies (*n* = 8) framed RTM as a causal inference and estimand identification problem, using tools such as DAGs and simulation to examine when baseline adjustment may mitigate bias and when it may introduce bias [2, 5, 7, 16, 37, 43, 50, 51]. Although some of these studies do not explicitly focus on RTM, they are included because they address the same underlying source of bias, namely, the dependence between baseline values and subsequent change, and clarify how analytical choices can produce misleading associations that are often attributed to RTM. Among these studies, there were explicit recommendations about when to adjust for baseline value of the outcome. Specifically, 4 studies noted that when the exposure starts before baseline or affects baseline outcome then routine baseline adjustment should be avoided [2, 7, 16, 50], and 4 studies noted that when the exposure starts after baseline or the baseline outcome determines the exposure then baseline adjustment might be appropriate [5, 7, 43, 51].

### Studies addressing ME

A total of 31 methodological studies addressed ME [20–22, 24, 25, 27, 29–31, 33, 39, 40, 42, 44–47, 54, 56–59, 61–63, 65, 70, 75, 76, 78, 80]. One of these studies [40] also examined additional methodological challenges, including confounding and missing data. Based on the dominant source of evidence supporting their conclusions, 11 studies were classified as methodological or conceptual [22, 25, 33, 39, 44, 58, 61, 65, 70, 76, 80], 11 as simulation studies [20, 21, 29, 42, 45, 54, 56, 57, 59, 63, 75], and 9 as applied examples using real data [24, 27, 30, 31, 40, 46, 47, 62, 78]. Most studies included some form of empirical illustration, with 25 of the 31 studies incorporating applications to real-world data [20–22, 24, 25, 27, 29–31, 33, 39, 40, 42, 44, 46, 47, 56–59, 61, 62, 65, 70, 78].

**Fig. 4:**
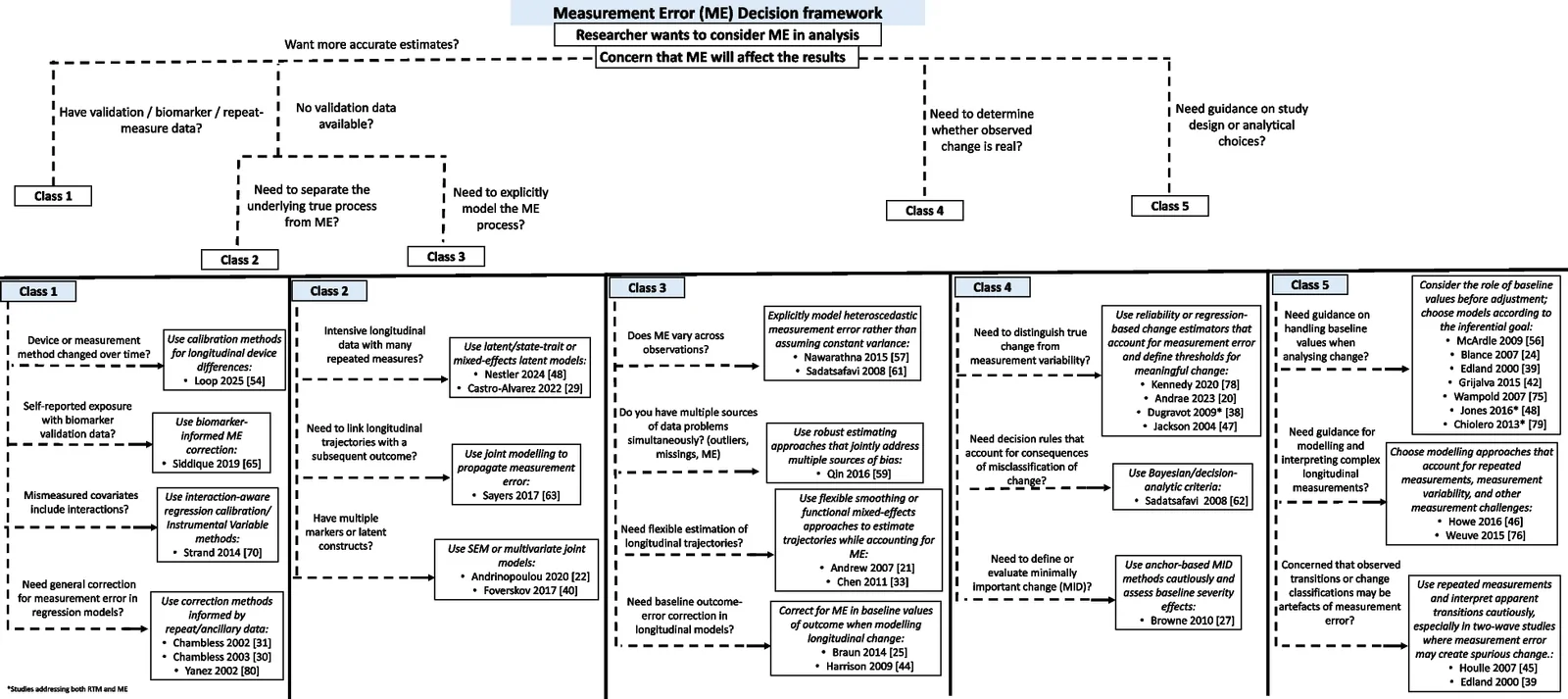
Decision framework for navigating methodological approaches to address measurement error (ME) in within-person change analysis Legend: Suggested classification of ME studies: class 1: calibration and validation-based correction; class 2: latent variable and joint modeling approaches; class 3: likelihood-based and flexible measurement error models; class 4: reliability-based change detection and decision frameworks; class 5: bias evaluation, design guidance, and methodological critique

Across studies, proposed strategies to address ME were grouped into five broad methodological classes (Fig. 4, Additional file 1: Table S2): (1) calibration and validation-based correction; (2) latent variable and joint modeling approaches; (3) likelihood-based and flexible measurement error models; (4) reliability-based change detection and decision frameworks; (5) bias evaluation, design guidance, and methodological critique.

#### Calibration and validation-based correction

Several studies (*n* = 6) proposed calibration-based approaches that use validation or ancillary data to correct for mismeasured variables [30, 31, 54, 65, 70, 80]. These methods include regression calibration, multiple imputation informed by validation data, biomarker or gold-standard validation sub-studies, instrumental-variable extensions, and method-of-moments bias correction using measurement-error estimates from replicate measurements. The aim of these approaches is to recover or approximate the underlying true values of mismeasured variables or to correct the resulting regression estimates for measurement-error bias.

#### Latent variable and joint modeling approaches

Another group of studies (*n* = 5) proposed latent-variable or joint modeling frameworks, including structural equation models, state–trait models, latent growth models, and multivariate joint longitudinal models [22, 29, 40, 58, 63]. These approaches assume that the observed measurements are imperfect indicators of an underlying true value. By modeling the unobserved true value separately from the measurement process, they aim to distinguish true change from measurement variability and account for ME throughout the longitudinal analysis.

#### Likelihood-based and flexible measurement error models

Several studies (*n* = 7) developed likelihood-based or flexible modeling approaches that incorporate measurement error directly into the statistical model [21, 25, 33, 44, 57, 59, 62]. These approaches included heteroscedastic measurement error models, robust estimating equations, functional mixed-effects models, and smoothing-based methods designed to account for complex error structures. Rather than correcting observed measurements before analysis, these methods model the magnitude and structure of measurement error during estimation, allowing uncertainty due to measurement error to be reflected in estimates of change and associations over time.

#### Reliability and regression-based change detection and decision frameworks

Some studies (*n* = 5) focused on frameworks for interpreting change in the presence of ME, rather than correcting regression estimates [20, 27, 47, 61, 78]. These approaches use reliability-based metrics such as the standard error of measurement, reliable change indices, Bayesian decision criteria, or regression-based approaches to distinguish meaningful change from measurement variability, particularly in clinical monitoring and patient-reported outcomes.

#### Bias evaluation and methodological guidance

Finally, a group of studies (*n* = 8) focused on evaluating or illustrating bias arising from ME, often through simulations or methodological critique [24, 39, 42, 45, 46, 56, 75, 76]. These studies examined how modeling choices such as baseline adjustment, anchoring baseline values as error-free, or using two-wave designs can introduce bias when ME is present, and provided guidance on appropriate design and analysis strategies.

### Studies addressing RTM and ME

Three studies [38, 48, 79] explicitly addressed RTM and ME while examining longitudinal associations. The first [38] used errors-in-variables regression informed by test–retest reliability to correct for ME and examined the implications of baseline adjustment. The second [48] used repeated pre-baseline HbA1c measurements and a bias-correction approach to assess the contribution of RTM and ME to associations between baseline HbA1c and treatment response. The third [79] used simulated longitudinal data to compare methods for assessing baseline-change associations under different levels of ME and between-individual variability in change, including Oldham’s method, Blomqvist’s method, repeated-measurement approaches, and linear random-effects modeling. Across these studies, the findings highlighted the importance of considering measurement processes when interpreting baseline-change associations. Further details on the approaches used, their strengths, limitations, and recommendations are provided in Additional file 1: Tables S1 and S2.

## Decision framework for method selection to take regression to the mean and measurement error into account

Based on our findings and proposed classification scheme, we developed a structured framework in the form of a decision tree to help navigate the methodological approaches identified for addressing RTM and ME (Figs. 3 and 4). This decision tree is guided by the main concerns that may come up during the analysis of longitudinal data. For example, if the primary concern is that an observed association between baseline values and subsequent change reflects RTM rather than a true underlying relationship, the RTM framework first distinguishes whether the objective is to quantify or correct for RTM, to use advanced distributional RTM estimation, or to model trajectories and/or repeated measures and maps these objectives to the relevant RTM methodological class. When the objective is to quantify or correct for RTM, the RTM framework maps to RTM class 1 (Fig. 3). If the specific question is how much change may be expected due to RTM alone, the framework identifies the studies by Tannenbaum et al. [71], Cameron et al. [28], and Veldhuizen et al. [74]. Additional file 1: Table S1 provides study-level information on the approaches used and, where reported, their strengths, limitations, and recommendations. Similarly, for ME, the framework first considers whether validation, biomarker, or repeat-measure data are available to improve estimation. When such data are available, the relevant approaches are mapped to class 1 and then to studies addressing the relevant measurement problem. For example, when a device or measurement method has changed over time, a relevant study is Loop et al. [54]. Study-level details are provided in Additional file 1: Table S2.

## Applied studies

The 102 included applied studies investigating indicators related to body weight and MetS covered a broad range of populations across the life course, including infants, children, adolescents, adults, and older adults. While many studies were conducted in general or apparently healthy populations, a substantial number focused on clinical groups such as individuals with obesity, diabetes, hypertension, HIV infection, psychiatric disorders, or those undergoing bariatric surgery (Additional file 2: Sheet S3).

**Fig. 5:**
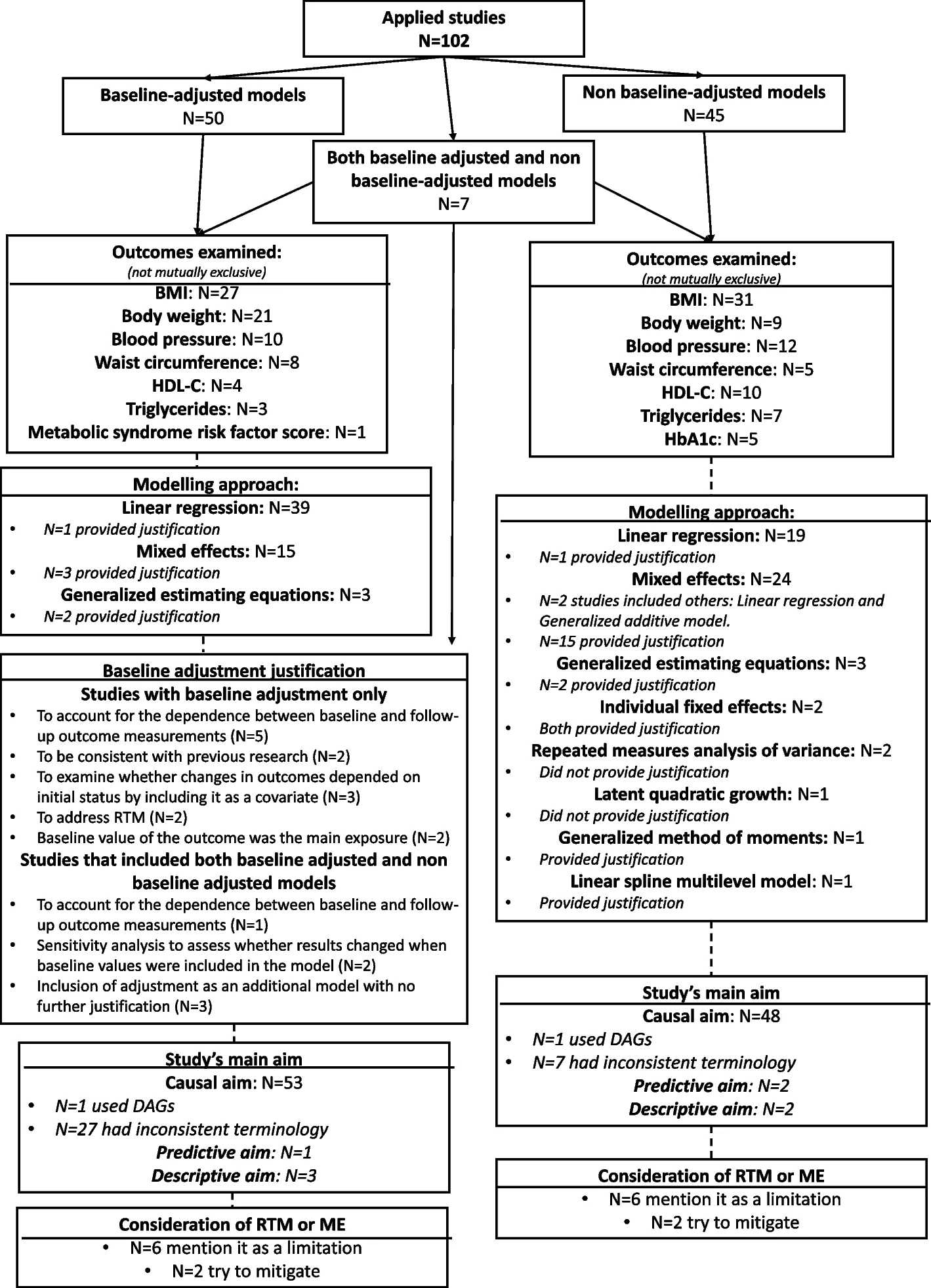
Distribution of study characteristics among applied studies according to baseline outcome adjustment approach

Of the 102 included, 50 (49%) [82, 84, 85, 87–91, 94, 96–98, 103, 105–107, 109, 113–116, 118, 119, 124, 126, 127, 129, 131, 133–136, 138–140, 148, 150–152, 157, 158, 160, 163, 167, 171, 172, 176–178, 180] used only models that adjusted for the baseline value of the outcome, 45 (44.1%) [81, 83, 86, 92, 95, 99–102, 104, 110, 111, 121, 123, 125, 128, 130, 132, 137, 141–147, 149, 153–156, 159, 161, 162, 164–166, 168, 169, 173–175, 179, 181, 182] used only models that did not adjust for baseline, and 7 (6.9%) [93, 108, 112, 117, 120, 122, 170] reported results from both baseline-adjusted and unadjusted approaches. The use of baseline adjustment varied across outcome types (Additional file 1: Table S3). Baseline-adjusted models were more common in studies examining body weight (69%) and WC (61%), whereas non-baseline-adjusted approaches predominated in studies of HDL-C (69%), triglycerides (70%), and HbA1c (100%). For BP, the distribution between approaches was more similar. Examination of analytical approaches by publication year did not reveal any clear temporal pattern in the use of baseline outcome adjustment (Additional file 1: Fig. S1). For descriptive analyses (Fig. 5), studies presenting both modeling strategies were included in both analytical groups.

### Studies that did not adjust for baseline value of the outcome

Among the 52 studies that did not adjust for the baseline value of the outcome, all used longitudinal designs with repeated outcome measurements ranging from two to 12 time points. The outcomes examined were not mutually exclusive, as several studies analyzed more than one outcome. BMI-related outcomes were the most frequently examined (*n* = 31 studies) [81, 83, 92, 93, 95, 99–101, 104, 108, 111, 112, 121, 122, 125, 128, 130, 132, 137, 141, 143–145, 153, 162, 164–166, 168, 179, 182], followed by BP (*n* = 12) [83, 102, 110, 120, 141, 149, 159, 161, 166, 174, 181, 182], HDL-C (*n* = 10), body weight (*n* = 9) [117, 137, 141, 145, 146, 154, 173, 175, 179], TG (*n* = 7) [104, 141, 145, 155, 156, 179, 181], HbA1c (*n* = 5) [86, 123, 147, 179, 182], and WC (*n* = 5) [83, 121, 122, 141, 143]. A wide range of exposures was investigated across studies, including behavioral, psychosocial, clinical, biological, and socioeconomic domains, including reproductive and metabolic factors, lifestyle characteristics, environmental influences, and clinical markers and treatments (Additional file 2: Sheet S3).

Mixed-effects models were the most commonly used approach (*n* = 24) [86, 92, 100, 102, 111, 120, 125, 130, 137, 142, 143, 146, 153, 155, 156, 159, 161, 162, 164–166, 173, 174, 179] used to analyze within-person change over time, including two studies [92, 174] that additionally applied alternative modeling strategies (generalized additive mixed models and ordinary least squares). Among these, 15 studies [86, 102, 125, 137, 142, 143, 146, 153, 156, 161, 162, 165, 173, 174, 179] provided a justification for the use of mixed-effects models, most commonly citing their ability to account for correlation between repeated measurements within individuals, accommodate unbalanced longitudinal data, and incorporate all available observations despite varying numbers and timing of follow-up measurements. Linear regression models were used in 19 studies [92, 93, 99, 101, 108, 110, 117, 121–123, 141, 144, 145, 154, 168–170, 175, 181], although only one study [101] explicitly justified this choice. Generalized estimating equations (GEEs) were used in three studies [81, 112, 147], two of which [81, 147] justified their use on the basis that GEEs account for within-individual correlation in repeated measurements and can handle unequal numbers of observations and irregular time intervals. A small number of studies used other modeling approaches, including individual fixed-effects models (*n* = 2) [83, 95], repeated-measures analysis of variance (*n* = 2) [104, 132], a latent quadratic growth model (*n* = 1) [128], the generalized method of moments (*n* = 1) [182], and linear spline multilevel models (*n* = 1) [149]. Only one of these [128] did not provide a justification for their model choice. The rest noted that these models exploit the longitudinal structure of the data or allow modeling of dynamic changes over time.

The majority of studies framed their analyses as estimating associations between exposures and subsequent change in outcomes. Specifically, 48 studies explicitly stated that their analyses aimed to estimate associations, indicating an explanatory or causal analytical framing. The use of formal causal tools was rare as only one study [166] employed DAGs to guide model specification. In seven studies [92, 104, 159, 162, 164, 165, 168], the interpretation of the analytical framework was unclear. These studies frequently used terminology such as “predict,” “predictor,” or “predicting” when describing regression results, although the analyses did not correspond to predictive modeling. This indicated some inconsistency between the terminology used to describe the analysis and its analytical aim. For example, Bond et al. [92] titled their study “*Lower cognitive functioning as a predictor of weight gain in bipolar disorder: a 12-month study*” and repeatedly referred to predictors in the text, while the stated objective was to examine the direction of the association between BMI and cognition, reflecting an explanatory rather than predictive aim. Two studies [101, 108] had a clearly predictive aim and used terminology consistent with this objective. Two studies [112, 154] had descriptive aims; however, one of these [154] used language suggesting explanatory interpretation despite the descriptive objective.

Consideration of ME or RTM was uncommon. Six studies [81, 83, 130, 141, 143, 175] mentioned ME as a potential limitation but did not implement analytical strategies to address it. Two studies both acknowledged and attempted to mitigate. The first [86] standardized laboratory measurements across examination waves to account for differences between and within laboratories consistent with class 1 methods for addressing measurement differences over time. The second [149] used age at peak height velocity as an objective measure of pubertal timing, noting that this approach reduces ME compared with self-reported assessments representing a class 5 approach focused on reducing ME through study design and measurement choice. The remaining studies neither mentioned nor addressed ME.

### Studies that adjusted for baseline value of the outcome

Among the 57 studies that adjusted for the baseline value of the outcome, all used longitudinal designs with repeated outcome measurements ranging from two to ten time points. The outcomes examined were not mutually exclusive, as several studies analyzed more than one outcome. BMI-related outcomes were the most frequently examined (*n* = 27) [85, 87, 89–91, 93, 94, 97, 103, 106, 108, 109, 112, 114–116, 118–120, 122, 126, 127, 150, 157, 163, 176, 178], followed by body weight (*n* = 21) [82, 84, 88, 91, 98, 116, 117, 124, 129, 131, 135, 138–140, 151, 158, 160, 167, 171, 172, 180], BP (*n* = 10) [85, 96, 113, 133, 134, 136, 148, 150, 152, 177], WC (*n* = 8) [85, 106, 107, 116, 122, 133, 134, 150], HDL-C (*n* = 4) [85, 133, 134, 170], TG (*n* = 3) [85, 133, 134], and MetS risk factor score (*n* = 1) [105]. A wide range of exposures across behavioral, clinical, biological, and socioeconomic domains was investigated (Additional file 2: Sheet S3).

Across studies, linear regression was the most frequently used approach (*n* = 39) [88, 91, 93, 94, 96–98, 103, 105, 107–109, 115–119, 122, 124, 126, 129, 131, 133–136, 139, 140, 150–152, 157, 158, 167, 170–172, 178, 180], although only one study [157] explicitly justified this choice. Linear mixed-effects models were used in 15 studies [82, 84, 85, 89, 90, 113, 114, 120, 127, 138, 148, 160, 163, 176, 177], three [90, 113, 177] of which provided a justification, while GEEs were used in three studies [87, 106, 112], two [87, 106] of which justified their use. The reasons for model choice were similar to those reported in studies without baseline adjustment.

Out of the 57 studies, 18 provided justification for adjusting for the baseline value of the outcome. The most common rationale was to account for the dependence between baseline and follow-up outcome measurements, allowing the effects of other covariates to be interpreted independently of initial outcome levels (*n* = 7) [109, 112, 115, 118, 124, 152, 157]. In two studies [87, 129], this choice was motivated by consistency with previous research. Some studies [119, 126, 135] justified baseline adjustment as a way to examine whether changes in outcomes depended on initial status by including it as a covariate. A small number of studies [122, 170] justified baseline adjustment as a sensitivity analysis to assess whether results changed when baseline values were included in the model. Two studies [148, 177] explicitly reported adjusting for baseline values to address RTM. Finally, in two studies [103, 120], the baseline value of the outcome was the main exposure.

The majority of studies framed their analyses as estimating associations between exposures and subsequent change in outcomes. Specifically, 53 of the 57 studies had an explanatory analytical aim and described associations between exposures and longitudinal changes in outcomes. However, interpretation was not always consistent with this framing, with similar inconsistencies in terminology observed in 27 studies [84, 87, 89, 91, 94, 96, 97, 103, 109, 114, 118, 126, 129, 131, 134, 135, 138, 151, 152, 157, 158, 163, 172, 176–178], as in studies without baseline adjustment. Only one study [98] employed DAGs to guide model specification. Three studies had primarily descriptive aims [112, 119, 167], and one study [108] had a clearly predictive objective.

Consideration of ME or RTM was also uncommon among studies that adjusted for baseline outcome values. Six studies [84, 89, 90, 115, 126, 127] mentioned either as a potential issue but did not implement analytical strategies to address it. Two studies [148, 177] explicitly attempted to address RTM using approaches that corresponded to several methodological classes identified in the review. Both studies justified adjustment for baseline outcome values as a strategy to address RTM, corresponding to class 6 approaches concerned with model selection and the role of baseline values. One study [148] additionally reduced measurement variability by requiring two consecutive baseline measurements before participant selection, consistent with class 5 study-design approaches. The other [177] averaged repeated BP measurements and estimated the expected magnitude of RTM using a published formula, corresponding to class 2 repeated-measurement approaches and class 1 RTM quantification methods, respectively. The remaining studies did not discuss these biases.

### Studies that reported baseline-adjusted and unadjusted models

Among the seven studies reporting both approaches [93, 108, 112, 117, 120, 122, 170], baseline adjustment was typically implemented in secondary or exploratory models. In most cases, authors examined whether additional adjustment for the baseline outcome altered effect estimates, explored potential mediation or interaction by baseline values, or assessed whether associations were independent of initial status. Across these studies, effect estimates were generally similar between baseline-adjusted and unadjusted models, and baseline adjustment was rarely explicitly justified from a methodological perspective. Only in one study [120] was the baseline value of the outcome considered as the primary exposure of interest where preoperative BMI was examined in relation to BMI measured repeatedly over time following bariatric surgery.

## Discussion

This scoping review synthesized methodological and applied literature on the analysis of within-person change in continuous outcomes in observational longitudinal health research. The methodological literature has extensively examined the challenges posed by RTM and ME and proposed a wide range of approaches to address these sources of bias, including analytical correction methods, model-based longitudinal approaches, causal and estimand-based frameworks, calibration methods, latent-variable models, and flexible ME models. By bringing together this heterogeneous body of work, our review provides a structured overview of the available methodological approaches. In addition, we developed a decision tree as a structured navigation tool to help researchers identify methods relevant to the specific sources of bias they seek to address. In contrast, the review of applied studies (using MetS components as example outcomes) revealed substantial heterogeneity in analytical practice. Approximately half of studies adjusted for baseline values of the outcome, while a similar proportion analyzed change without baseline adjustment, and only a small minority presented both approaches. Justification for modeling choices was uncommon, the use of formal causal tools was rare, and ME was seldom discussed or addressed analytically. Because the applied component was intentionally restricted to studies examining selected anthropometric and metabolic outcomes, the observed distribution of analytical approaches should not be interpreted as representative of all areas of observational longitudinal health research.

### Methods studies

The methodological literature addressing RTM proposed a range of approaches like analytical correction formulas, longitudinal modeling frameworks, alternative estimators designed to avoid mathematical coupling, likelihood-based approaches, and design-stage strategies. A number of studies also framed RTM within a causal inference perspective, emphasizing that the role of baseline adjustment depends on the underlying causal structure and the timing of exposure relative to baseline measurement. Despite differences in analytical approach, several common themes emerged across these studies. Many emphasized that RTM arises from the combination of measurement variability, study design, and modeling choices, and therefore cannot always be addressed through statistical adjustment alone. Across studies, there was broad agreement that repeated measurements and reliable instruments improve the ability to distinguish true change from statistical artifact, and that analytical strategies should be aligned with the research question and causal structure. Importantly, several studies cautioned that baseline adjustment does not automatically remove RTM and may introduce bias in some contexts, particularly when exposures influence baseline outcome values. One study [5] explicitly recommends avoiding change-score analyses in observational settings due to their potential to yield misleading causal estimates, whereas other studies [2, 7, 16, 37, 43, 50, 51] emphasize that both change-score and baseline-adjusted approaches may be appropriate depending on the estimand of interest and underlying causal assumptions. For example, when the exposure precedes the baseline value of the outcome or affects the baseline then adjustment for baseline is not recommended. This divergence reflects broader differences in how change is conceptualized and highlights the importance of aligning analytical choices with clearly defined causal questions. By synthesizing these diverse approaches, our review demonstrates that the methodological literature does not support a single universally preferred strategy for addressing RTM. Instead, the appropriate approach depends on the source of bias, the study design, the available data, and the causal question being addressed. To facilitate navigation of this heterogeneous literature, we grouped methodological approaches into broad categories and developed a structured framework to map the methods identified in the literature to specific analytical challenges (Fig. 3).

The methodological literature addressing ME similarly proposed a range of approaches, including calibration and validation-based corrections, latent variable and joint modeling frameworks, likelihood-based models that explicitly parameterize ME processes, and reliability-based approaches for interpreting change. A further group of studies focused on evaluating bias arising from ME and providing guidance on study design and analytical strategies. Across these studies, a common theme was that ME can substantially distort estimates of change and associations with baseline values if it is ignored or treated as negligible. Many approaches emphasized the importance of distinguishing the underlying signal from measurement variability, particularly in longitudinal settings where repeated assessments are used to infer individual trajectories. Although the proposed methods differ in complexity and data requirements, they have several shared recommendations. These include the use of validation or repeat measurements where possible, careful consideration of the measurement process when specifying statistical models, and caution against treating baseline measurements as error-free. Several studies also emphasized that reliance on two-wave designs limits the ability to distinguish true change from measurement variability and recommended the use of multiple measurement occasions when feasible. Collectively, these findings highlight that addressing ME requires attention not only to statistical correction but also to study design, measurement quality, and transparent reporting of analytical assumptions. Our synthesis shows that available methods span a broad spectrum of complexity and data requirements, ranging from relatively simple calibration-based corrections to more advanced latent-variable and likelihood-based approaches. By organizing this literature into a structured framework, the review provides an evidence-mapping tool for navigating the methodological options available to researchers seeking to address ME in longitudinal analyses of change (Fig. 4).

Based on these findings, the developed decision frameworks help researchers navigate the available methods for addressing RTM and ME in analyses of within-person change (Figs. 3 and 4). Rather than recommending a single analytical approach, each framework organizes the identified methods around a series of questions based on the researcher’s specific analytical concern and the available data. These questions map the methodological classes identified separately for RTM and ME and the approaches within each class. For example, a researcher concerned that an observed change may reflect RTM can use Fig. 3 to distinguish between approaches for quantifying or correcting expected RTM, modeling longitudinal trajectories using repeated measurements, or applying more advanced distributional RTM estimation. Similarly, Fig. 4 organizes approaches according to the nature of the ME problem and the availability of information such as validation, biomarker, or repeat-measure data, and maps these to the relevant ME methodological classes and approaches within each class. The frameworks therefore provide a structured entry point into a heterogeneous methodological literature, while the Results section and supplementary tables provide further details on the individual methods.

### Applied studies

In contrast to the breadth of methodological work addressing RTM and ME, the applied literature showed limited evidence that these methodological considerations are routinely incorporated into practice.

Among the applied studies (using MetS indicators and body weight as example outcomes), a wide range of exposures and populations were examined, reflecting the broad use of longitudinal observational data to investigate determinants of change in metabolic and anthropometric outcomes. Mixed-effects models were the most frequently used approach in studies that did not adjust for baseline outcome values, whereas linear regression models were most common in studies that adjusted for baseline. Despite the diversity of statistical models employed, explicit justification for the chosen modeling strategy was uncommon across both groups. Most studies framed their analyses as examining associations between exposures and subsequent changes in outcomes, although the terminology used in reporting results was often inconsistent with this explanatory aim. This inconsistency also supports our intentional inclusion of broad terms such as “predictor” and “risk factor” in the search strategy, as restricting the search according to strict causal terminology could have resulted in relevant studies being missed.

Consideration of RTM and ME was uncommon across the applied literature. Although a small number of studies discussed these issues, few attempted to address them analytically. Notably, some studies justified adjustment for baseline outcome values as a strategy to control RTM. However, the methodological literature reviewed here suggests that the role of baseline adjustment remains debated. In particular, several studies highlighted that baseline-adjusted analyses may themselves be biased when baseline outcome values are measured with error, and that associations between baseline values and subsequent change can be distorted by RTM and mathematical coupling. These findings suggest that baseline adjustment should not be viewed as a universally appropriate solution to RTM.

An interesting difference between the two analytical approaches concerned the distribution of outcomes examined. Studies adjusting for baseline outcome values more frequently focused on weight-related outcomes such as body weight and BMI, whereas studies that did not adjust for baseline included a broader range of metabolic indicators, including blood pressure and lipid measures. One possible explanation is that in research on weight change, adjustment for baseline weight or BMI is often viewed as necessary to account for differences in initial body size and to facilitate interpretation of subsequent change. However, baseline adjustment was rarely justified explicitly in methodological terms, and in most studies the rationale for including baseline outcome values was not clearly stated. As a result, it was often unclear whether baseline adjustment reflected a deliberate analytical choice based on the underlying causal question or a routine modeling practice adopted without explicit consideration of its assumptions and implications.

An additional issue encountered during study selection concerned the interpretation of longitudinal mixed-effects models. Some studies used mixed models with repeated outcome measurements but without including an interaction between exposure and time, while interpreting the resulting coefficients as representing exposure-specific within-person change. In such models, the estimated associations reflect average differences in outcome levels between exposure groups across time rather than differences in trajectories or rates of change. Because these models do not estimate exposure-specific change over time, studies using this approach were excluded from the review (e.g., [183–185]). This highlights a potential source of confusion in the applied literature and underscores the importance of clearly specifying how change is defined and estimated in longitudinal analyses.

### Strengths and limitations

This review has several strengths. By synthesizing both methodological and applied studies, it provides a comprehensive overview of how within-person change is currently analyzed in observational health research and how these practices relate to the broader methodological literature on RTM and ME. We also systematically organized a heterogeneous methodological literature into a coherent framework to help researchers navigate and identify methodological approaches relevant to the specific sources of bias they seek to address. The review also focused on commonly studied metabolic and anthropometric outcomes, allowing for clearer comparison of analytical approaches across studies within a relatively coherent research domain. In addition, the systematic search strategy, duplicate screening, and data extraction strengthened the transparency and rigor of the review. However, several limitations should be considered. To focus our research, we used components of MetS and body weight as an example for the applied studies; therefore, the applied component was restricted to a subset of metabolic and anthropometric outcomes, which may limit the generalizability of the findings to other areas of observational health research. However, MetS and its components remain highly prevalent conditions that cause substantial burden of disease globally, causing a high interest into studying its natural courses and determinants [186, 187]. The classification of analytical approaches necessarily simplified complex modeling strategies and relied on the information reported in the original articles, which was sometimes limited. In addition, the methodological literature was highly heterogeneous with respect to study designs, analytical objectives, and sources of evidence, and the grouping of methods into broad categories inevitably involved some degree of judgment. Finally, because the review aimed to characterize methodological recommendations and analytical practices rather than evaluate the performance of specific statistical methods, it does not provide empirical comparisons of the relative performance of different modeling approaches.

## Conclusions

In summary, this review highlights the heterogeneity in how within-person change in continuous outcomes is analyzed in observational health research and a limited alignment between the methodological literature addressing RTM and ME and the common analytical practices. Across methodological studies, a consistent message emerged that biases in analyses of change often arise from the interaction of measurement variability, study design, and modeling choices. These findings underscore the importance of clearly specifying the research question and estimand, carefully considering the timing of exposure relative to baseline outcome measurement when deciding whether to adjust for baseline values, and avoiding the assumption that baseline measurements are error-free. We also developed a decision tree to help researchers navigate the methodological approaches identified for taking RTM or ME into account in longitudinal data analyses. Greater use of repeated measurements, transparent reporting of analytical decisions, and modeling strategies aligned with both the causal structure and the measurement properties of the data may help improve the validity and interpretability of studies examining within-person change.

## Supplementary Information

Supplementary Material 1.

Supplementary Material 2.

## Data Availability

No datasets were generated or analysed during the current study.

## Abbreviations

NAKO: German National Cohort
RTM: Regression to the mean
ME: Measurement error
DAG: Directed acyclic graph
HDL-C: High-density lipoprotein cholesterol
HbA1c: Hemoglobin A1c
TG: Triglycerides
WC: Waist circumference
BP: Blood pressure
PRISMA: Preferred Reporting Items for Systematic Review and Meta-Analysis

## References

[1] Peters A, Peters A, Greiser KH, Göttlicher S, Ahrens W, Albrecht M, et al. Framework and baseline examination of the German National Cohort (NAKO). Eur J Epidemiol. 2022;37(10):1107–24. 10.1007/s10654-022-00890-5 PMC9581448 36260190

[2] Glymour MM, Weuve J, Berkman LF, Kawachi I, Robins JM. When is baseline adjustment useful in analyses of change? An example with education and cognitive change. Am J Epidemiol. 2005;162(3):267–78. 10.1093/aje/kwi187 15987729

[3] Sorjonen K, Ingre M, Nilsonne G, Melin B. Dangers of including outcome at baseline as a covariate in latent change score models: results from simulations and empirical re-analyses. Heliyon. 2023;9(5):e15746. 10.1016/j.heliyon.2023.e15746 PMC10160499 37153390

[4] Hammerton G, Munafò MR. Causal inference with observational data: the need for triangulation of evidence. Psychol Med. 2021;51(4):563–78. 10.1017/S0033291720005127 PMC8020490 33682654

[5] Tennant PWG, Arnold KF, Ellison GTH, Gilthorpe MS. Analyses of ‘change scores’ do not estimate causal effects in observational data. Int J Epidemiol. 2022;51(5):1604–15. 10.1093/ije/dyab050 PMC9557845 34100077

[6] Tu YK, Gilthorpe MS. Revisiting the relation between change and initial value: a review and evaluation. Stat Med. 2007;26(2):443–57. 10.1002/sim.2538 16526009

[7] Dukes O, Shahn Z, Renson A. Change scores and baseline adjustment: splitting the difference (in differences). Int J Epidemiol. 2025;54(3):dyaf042. 10.1093/ije/dyaf042 40358090

[8] Senn S. Change from baseline and analysis of covariance revisited. Stat Med. 2006;25(24):4334–44. 10.1002/sim.2682 16921578

[9] Yang L, Tsiatis A. Efficiency study of estimators for a treatment effect in a pretest-posttest trial. Am Stat. 2001;55:314–21.

[10] Archie JP Jr. Mathematic coupling of data: a common source of error. Ann Surg. 1981;193(3):296–303. 10.1097/00000658-198103000-00008 PMC1345065 7212790

[11] Hayes R. Methods for assessing whether change depends on initial value. Stat Med. 1988;7(9):915–27. 10.1002/sim.4780070903 3175391

[12] Kirkwood BR, Sterne JA. Essential medical statistics. John Wiley & Sons; 2010.

[13] Blomqvist N. On the relation between change and initial value. J Am Stat Assoc. 1977;72(360a):746–9.

[14] Healy M. Some problems of repeated measurements. Perspectives in medical statistics, Bithell JF, Coppi R (eds) Academic Press: London. 1981.

[15] Oldham P. A note on the analysis of repeated measurements of the same subjects. J Chronic Dis. 1962;15(10):969–77. 10.1016/0021-9681(62)90116-9 13939936

[16] Glymour MM. Commentary: modelling change in a causal framework. Int J Epidemiol. 2022;51(5):1615–21. 10.1093/ije/dyac151 PMC9558068 35900266

[17] Tricco AC, Lillie E, Zarin W, O’Brien KK, Colquhoun H, Levac D, et al. PRISMA extension for scoping reviews (PRISMA-ScR): checklist and explanation. Ann Intern Med. 2018;169(7):467–73. 10.7326/M18-0850 30178033

[18] Bahor Z, Liao J, Currie G, Ayder C, Macleod M, McCann SK, et al. Development and uptake of an online systematic review platform: the early years of the CAMARADES Systematic Review Facility (SyRF). BMJ Open Sci. 2021;5(1): e100103. 10.1136/bmjos-2020-100103 PMC8647599 35047698

[19] Team TE. Endnote. EndNote 2025 ed. Philadelphia, PA: Clarivate; 2013.

[20] Andrae DA, Foster B, Peipert JD. Comparison of raw and regression approaches to capturing change on patient-reported outcome measures. Qual Life Res. 2023;32(5):1381–90. 10.1007/s11136-022-03196-x 36136261

[21] Andrew ME, Li S, Fekedulegn D, Dorn J, Joseph PN, Violanti J, et al. Estimation of the maximum flow-mediated brachial artery response using local regression methods. Physiol Meas. 2007;28(10):1213–24. 10.1088/0967-3334/28/10/007 17906389

[22] Andrinopoulou ER, Clancy JP, Szczesniak RD. Multivariate joint modeling to identify markers of growth and lung function decline that predict cystic fibrosis pulmonary exacerbation onset. BMC Pulm Med. 2020;20(1):142. 10.1186/s12890-020-1177-z PMC7236487 32429862

[23] Barnett AG, van der Pols JC, Dobson AJ. Regression to the mean: what it is and how to deal with it. Int J Epidemiol. 2005;34(1):215–20. 10.1093/ije/dyh299 15333621

[24] Blance A, Tu YK, Baelum V, Gilthorpe MS. Statistical issues on the analysis of change in follow-up studies in dental research. Community Dent Oral Epidemiol. 2007;35(6):412–20. 10.1111/j.1600-0528.2007.00407.x 18039282

[25] Braun J, Held L, Ledergerber B. Accounting for baseline differences and measurement error in the analysis of change over time. Stat Med. 2014;33(1):2–16. 10.1002/sim.5910 23900718

[26] Brown R, Hailu EM, Needham BL, Roux AD, Seeman TE, Lin J, et al. Neighborhood social environment and changes in leukocyte telomere length: the Multi-Ethnic Study of Atherosclerosis (MESA). Health Place. 2021;67:102488. 10.1016/j.healthplace.2020.102488 33276262

[27] Browne JP, van der Meulen JH, Lewsey JD, Lamping DL, Black N. Mathematical coupling may account for the association between baseline severity and minimally important difference values. J Clin Epidemiol. 2010;63(8):865–74. 10.1016/j.jclinepi.2009.10.004 20172689

[28] Cameron N, Preece MA, Cole TJ. Catch-up growth or regression to the mean? Recovery from stunting revisited. Am J Hum Biol. 2005;17(4):412–7. 10.1002/ajhb.20408 15981181

[29] Castro-Alvarez S, Tendeiro JN, Meijer RR, Bringmann LF. Using structural equation modeling to study traits and states in intensive longitudinal data. Psychol Methods. 2022;27(1):17–43. 10.1037/met0000393 34014719

[30] Chambless LE, Davis V. Analysis of associations with change in a multivariate outcome variable when baseline is subject to measurement error. Stat Med. 2003;22(7):1041–67. 10.1002/sim.1352 12652553

[31] Chambless LE, Folsom AR, Davis V, Sharrett R, Heiss G, Sorlie P, et al. Risk factors for progression of common carotid atherosclerosis: the Atherosclerosis Risk in Communities Study, 1987–1998. Am J Epidemiol. 2002;155(1):38–47. 10.1093/aje/155.1.38 11772783

[32] Chapurlat RD, Blackwell T, Bauer DC, Cummings SR. Changes in biochemical markers of bone turnover in women treated with raloxifene: influence of regression to the mean. Osteoporos Int. 2001;12(12):1006–14. 10.1007/s001980170009 11846325

[33] Chen H, Wang Y. A penalized spline approach to functional mixed effects model analysis. Biometrics. 2011;67(3):861–70. 10.1111/j.1541-0420.2010.01524.x 21155747

[34] Choy A, Stanton E, Gegechkori N, Gibson K, Strzeciwilk M, Lam J, et al. Premature ventricular complexes after catheter ablation for atrial fibrillation: accounting for regression to the mean. J Cardiovasc Electrophysiol. 2024;35(8):1688–90. 10.1111/jce.16333 38845147

[35] Cole TJ, Wright CM. A chart to predict adult height from a child’s current height. Ann Hum Biol. 2011;38(6):662–8. 10.3109/03014460.2011.598189 21767107

[36] Cysique LA, Vaida F, Letendre S, Gibson S, Cherner M, Woods SP, et al. Dynamics of cognitive change in impaired HIV-positive patients initiating antiretroviral therapy. Neurology. 2009;73(5):342–8. 10.1212/WNL.0b013e3181ab2b3b PMC2725933 19474412

[37] Daw JR, Hatfield LA. Matching and regression to the mean in difference-in-differences analysis. Health Serv Res. 2018;53(6):4138–56. 10.1111/1475-6773.12993 PMC6232412 29957834

[38] Dugravot A, Guéguen A, Kivimaki M, Vahtera J, Shipley M, Marmot MG, et al. Socioeconomic position and cognitive decline using data from two waves: what is the role of the wave 1 cognitive measure? J Epidemiol Community Health. 2009;63(8):675–80. 10.1136/jech.2008.081281 PMC2789968 19406741

[39] Edland SD. Blomqvist revisited: how and when to test the relationship between level and longitudinal rate of change. Stat Med. 2000;19(11–12):1441–52. 10.1002/(sici)1097-0258(20000615/30)19:11/12<1441::aid-sim436>3.0.co;2-h 10844709

[40] Foverskov E, Mortensen EL, Holm A, Pedersen JLM, Osler M, Lund R. Socioeconomic position across the life course and cognitive ability later in life: the importance of considering early cognitive ability. J Aging Health. 2019;31(6):947–66. 10.1177/0898264317742810 29254458

[41] Gmel G, Wicki M, Rehm J, Heeb JL. Estimating regression to the mean and true effects of an intervention in a four-wave panel study. Addiction. 2008;103(1):32–41. 10.1111/j.1360-0443.2007.02034.x 17995991

[42] Grijalva CG, Roumie CL, Murff HJ, Hung AM, Beck C, Liu X, et al. The role of matching when adjusting for baseline differences in the outcome variable of comparative effectiveness studies. J Comp Eff Res. 2015;4(4):341–9. 10.2217/cer.15.16 PMC4699664 26274795

[43] Gustavson K, Borren I. Bias in the study of prediction of change: a Monte Carlo simulation study of the effects of selective attrition and inappropriate modeling of regression toward the mean. BMC Med Res Methodol. 2014;14:133. 10.1186/1471-2288-14-133 PMC4298063 25519494

[44] Harrison L, Dunn DT, Green H, Copas AJ. Modelling the association between patient characteristics and the change over time in a disease measure using observational cohort data. Stat Med. 2009;28(26):3260–75. 10.1002/sim.3725 19768690

[45] Houle TT, Turner DP, Smitherman TA, Penzien DB, Lipton RB. Influence of random measurement error on estimated rates of headache chronification and remission. Headache. 2013;53(6):920–9. 10.1111/head.12125 PMC9128797 23721239

[46] Howe LD, Tilling K, Matijasevich A, Petherick ES, Santos AC, Fairley L, et al. Linear spline multilevel models for summarising childhood growth trajectories: a guide to their application using examples from five birth cohorts. Stat Methods Med Res. 2016;25(5):1854–74. 10.1177/0962280213503925 PMC4074455 24108269

[47] Jackson AS, Kampert JB, Barlow CE, Morrow JR Jr, Church TS, Blair SN. Longitudinal changes in cardiorespiratory fitness: measurement error or true change? Med Sci Sports Exerc. 2004;36(7):1175–80. 10.1249/01.mss.0000132269.26126.3b 15235321

[48] Jones AG, Lonergan M, Henley WE, Pearson ER, Hattersley AT, Shields BM. Should studies of diabetes treatment stratification correct for baseline HbA1c? PLoS ONE. 2016;11(4):e0152428. 10.1371/journal.pone.0152428 PMC4822872 27050911

[49] Kelly C, Price TD. Correcting for regression to the mean in behavior and ecology. Am Nat. 2005;166(6):700–7. 10.1086/497402 16475086

[50] Kramer MS, Zhang X, Bin Aris I, Dahhou M, Naimi A, Yang S, et al. Methodological challenges in studying the causal determinants of child growth. Int J Epidemiol. 2016;45(6):2030–7. 10.1093/ije/dyw090 PMC5841613 27297676

[51] Lepage B, Lamy S, Dedieu D, Savy N, Lang T. Estimating the causal effect of an exposure on change from baseline using directed acyclic graphs and path analysis. Epidemiology. 2015;26(1):122–9. 10.1097/EDE.0000000000000192 25401453

[52] Leung M, Bassani DG, Racine-Poon A, Goldenberg A, Ali SA, Kang G, et al. Conditional random slope: a new approach for estimating individual child growth velocity in epidemiological research. Am J Hum Biol. 2017. 10.1002/ajhb.23009. PMC5599979 28429467

[53] Li Y. Inferring meaningful change in quality of life with posterior predictive distribution: an alternative to standard error of measurement. Qual Life Res. 2023;32(5):1391–400. 10.1007/s11136-022-03239-3 PMC10202216 36083421

[54] Loop MS, Lotspeich SC, Garcia TP, Meyer ML. Should regression calibration or multiple imputation be used when calibrating different devices in a longitudinal study? Am J Epidemiol. 2025;194(1):295–301. 10.1093/aje/kwae169 PMC11735970 38957970

[55] Marsden J, Eastwood B, Wright C, Bradbury C, Knight J, Hammond P. How best to measure change in evaluations of treatment for substance use disorder. Addiction. 2011;106(2):294–302. 10.1111/j.1360-0443.2010.03143.x 20925686

[56] McArdle PF, Whitcomb BW. Improper adjustment for baseline in genetic association studies of change in phenotype. Hum Hered. 2009;67(3):176–82. 10.1159/000181156 PMC2868920 19077436

[57] Nawarathna LS, Choudhary PK. A heteroscedastic measurement error model for method comparison data with replicate measurements. Stat Med. 2015;34(7):1242–58. 10.1002/sim.6424 25614299

[58] Nestler S, Blozis SA. A latent variable mixed-effects location scale model that also considers between-person differences in the autocorrelation. Stat Med. 2024;43(1):89–101. 10.1002/sim.9943 37927154

[59] Qin G, Zhang J, Zhu Z, Fung W. Robust estimation of partially linear models for longitudinal data with dropouts and measurement error. Stat Med. 2016;35(29):5401–16. 10.1002/sim.7062 27460857

[60] Ripatti S, Mäkelä P. Conditional models accounting for regression to the mean in observational multi-wave panel studies on alcohol consumption. Addiction. 2008;103(1):24–31. 10.1111/j.1360-0443.2007.02033.x 17995992

[61] Sadatsafavi M, Moayyeri A, Wang L, Leslie WD. Optimal decision criterion for detecting change in bone mineral density during serial monitoring: a Bayesian approach. Osteoporos Int. 2008;19(11):1589–96. 10.1007/s00198-008-0615-1 18427707

[62] Sadatsafavi M, Moayyeri A, Wang L, Leslie WD. Heteroscedastic regression analysis of factors affecting BMD monitoring. J Bone Miner Res. 2008;23(11):1842–9. 10.1359/jbmr.080602 18925862

[63] Sayers A, Wylde V, Lenguerrand E, Gooberman-Hill R, Dawson J, Beard D, et al. A unified multi-level model approach to assessing patient responsiveness including; return to normal, minimally important differences and minimal clinically important improvement for patient reported outcome measures. BMJ Open. 2017;7(7):e014041. 10.1136/bmjopen-2016-014041 PMC5577889 28733296

[64] Schmidt MI, Bracco P, Canhada S, Guimarães JMN, Barreto SM, Chor D, et al. Regression to the mean contributes to the apparent improvement in glycemia 3.8 years after screening: the ELSA-Brasil study. Diabetes Care. 2021;44(1):81–8. 10.2337/dc20-2030 PMC7783938 33444159

[65] Siddique J, Daniels MJ, Carroll RJ, Raghunathan TE, Stuart EA, Freedman LS. Measurement error correction and sensitivity analysis in longitudinal dietary intervention studies using an external validation study. Biometrics. 2019;75(3):927–37. 10.1111/biom.13044 PMC7593985 30724332

[66] Skog OJ, Rossow I. Flux and stability: individual fluctuations, regression towards the mean and collective changes in alcohol consumption. Addiction. 2006;101(7):959–70. 10.1111/j.1360-0443.2006.01492.x 16771888

[67] Soer R, Reneman MF, Mierau JO, Schiphorst Preuper HR, Stegeman P, Speijer B, et al. Can we change health care costs in patients with complex back pain?: results from a 5-year before and after study. Spine (Phila Pa 1976). 1976;45(20):1443–50. 10.1097/BRS.0000000000003550 PMC7515478 32502071

[68] Sorjonen K, Melin B. Prospective associations between social support and posttraumatic stress disorder may be spurious: a re-meta-analysis of longitudinal studies and a simulation. J Affect Disord. 2023;340:174–80. 10.1016/j.jad.2023.08.014 37557992

[69] Sorjonen K, Nilsonne G, Ingre M, Melin B. Regression to the mean in latent change score models: an example involving breastfeeding and intelligence. BMC Pediatr. 2022;22(1):283. 10.1186/s12887-022-03349-4 PMC9109323 35578205

[70] Strand M, Sillau S, Grunwald GK, Rabinovitch N. Regression calibration for models with two predictor variables measured with error and their interaction, using instrumental variables and longitudinal data. Stat Med. 2014;33(3):470–87. 10.1002/sim.5904 PMC4104685 23901041

[71] Tannenbaum C, Barrett-Connor E, Laughlin GA, Platt RW. A longitudinal study of dehydroepiandrosterone sulphate (DHEAS) change in older men and women: the Rancho Bernardo Study. Eur J Endocrinol. 2004;151(6):717–25. 10.1530/eje.0.1510717 15588238

[72] Tu YK, Baelum V, Gilthorpe MS. The problem of analysing the relationship between change and initial value in oral health research. Eur J Oral Sci. 2005;113(4):271–8. 10.1111/j.1600-0722.2005.00228.x 16048518

[73] Umair M, Khan M, Olivier J. Accounting for regression to the mean under the bivariate t-distribution. Stat Methods Med Res. 2024;33(9):1624–36. 10.1177/09622802241267808 39118352

[74] Veldhuizen S, Bedard C, King-Dowling S, Barratt J, Kwan M, Cairney J. Stability of M-ABC-2-measured motor functioning in early childhood: evidence from a large prospective cohort. Hum Mov Sci. 2023;87:103037. 10.1016/j.humov.2022.103037 36442296

[75] Wampold BE, Bolt DM. The consequences of “anchoring” in longitudinal multilevel models: bias in the estimation of patient variability and therapist effects. Psychother Res. 2007;17(5):509–14.

[76] Weuve J, Proust-Lima C, Power MC, Gross AL, Hofer SM, Thiébaut R, et al. Guidelines for reporting methodological challenges and evaluating potential bias in dementia research. Alzheimers Dement. 2015;11(9):1098–109. 10.1016/j.jalz.2015.06.1885 PMC4655106 26397878

[77] Zhang X, Tomblin JB. Explaining and controlling regression to the mean in longitudinal research designs. J Speech Lang Hear Res. 2003;46(6):1340–51. 10.1044/1092-4388(2003/104) 14700359

[78] Kennedy DL, Kemp HI, Wu C, Ridout DA, Rice ASC. Determining real change in conditioned pain modulation: a repeated measures study in healthy volunteers. J Pain. 2020;21(5–6):708–21. 10.1016/j.jpain.2019.09.010 31715262

[79] Chiolero A, Paradis G, Rich B, Hanley JA. Assessing the relationship between the baseline value of a continuous variable and subsequent change over time. Front Public Health. 2013;1:29. 10.3389/fpubh.2013.00029 PMC3854983 24350198

[80] Yanez ND, Kronmal RA, Shemanski LR, Psaty BM, for the Cardiovascular Health S. A regression model for longitudinal change in the presence of measurement error. Ann Epidemiol. 2002;12(1):34–8. 10.1016/s1047-2797(01)00280-0 11750238

[81] Abrams B, Heggeseth B, Rehkopf D, Davis E. Parity and body mass index in US women: a prospective 25-year study. Obesity (Silver Spring). 2013;21(8):1514–8. 10.1002/oby.20503 PMC3752308 23630108

[82] Abu-Rumaileh M, Haddad RA, Yosef M, Esfandiari NH, Kraftson A, Khairi S, et al. Impact of nonalcoholic fatty liver disease (NAFLD) on weight loss after bariatric surgery. Obes Surg. 2023;33(12):3814–28. 10.1007/s11695-023-06865-z PMC12330159 37940737

[83] Adjaye-Gbewonyo K, Kawachi I, Subramanian SV, Avendano M. Income inequality and cardiovascular disease risk factors in a highly unequal country: a fixed-effects analysis from South Africa. Int J Equity Health. 2018;17(1):31. 10.1186/s12939-018-0741-0 PMC5839065 29510733

[84] Alebel A, Demant D, Petrucka PM, Sibbritt D. Weight change after antiretroviral therapy initiation among adults living with HIV in Northwest Ethiopia: a longitudinal data analysis. BMJ Open. 2022;12(2):e055266. 10.1136/bmjopen-2021-055266 PMC8808440 35105589

[85] Andersen LB, Wedderkopp N, Kristensen P, Moller NC, Froberg K, Cooper AR. Cycling to school and cardiovascular risk factors: a longitudinal study. J Phys Act Health. 2011;8(8):1025–33. 10.1123/jpah.8.8.1025 22039135

[86] Azevedo Da Silva M, Dugravot A, Balkau B, Roussel R, Fumeron F, Elbaz A, et al. Antidepressant medication use and trajectories of fasting plasma glucose, glycated haemoglobin, β-cell function and insulin sensitivity: a 9-year longitudinal study of the D.E.S.I.R. cohort. Int J Epidemiol. 2015;44(6):1927–40. 10.1093/ije/dyv153 26245205

[87] Baker JF, Cannon GW, Ibrahim S, Haroldsen C, Caplan L, Mikuls TR. Predictors of longterm changes in body mass index in rheumatoid arthritis. J Rheumatol. 2015;42(6):920–7. 10.3899/jrheum.141363 PMC4826749 25834210

[88] Barr SI. Calcium and body fat in peripubertal girls: cross-sectional and longitudinal observations. Obesity Silver Spring. 2007;15(5):1302–10. 10.1038/oby.2007.152 17495207

[89] Bel-Serrat S, Heinen MM, Mehegan J, O’Brien S, Eldin N, Murrin CM, et al. Predictors of weight status in school-aged children: a prospective cohort study. Eur J Clin Nutr. 2019;73(9):1299–306. 10.1038/s41430-018-0359-8 30446761

[90] Berkey CS, Rockett HR, Willett WC, Colditz GA. Milk, dairy fat, dietary calcium, and weight gain: a longitudinal study of adolescents. Arch Pediatr Adolesc Med. 2005;159(6):543–50. 10.1001/archpedi.159.6.543 15939853

[91] Bhat YR, Rolls BJ, Wilson SJ, Rose E, Geier CF, Fuchs B, et al. Eating in the absence of hunger is a stable predictor of adiposity gains in middle childhood. J Nutr. 2024;154(12):3726–39. 10.1016/j.tjnut.2024.10.008 PMC11662233 39393498

[92] Bond DJ, Torres IJ, Lee SS, Kozicky JM, Silveira LE, Dhanoa T, et al. Lower cognitive functioning as a predictor of weight gain in bipolar disorder: a 12-month study. Acta Psychiatr Scand. 2017;135(3):239–49. 10.1111/acps.12674 27995622

[93] Callaway LK, McIntyre HD, O’Callaghan M, Williams GM, Najman JM, Lawlor DA. The association of hypertensive disorders of pregnancy with weight gain over the subsequent 21 years: findings from a prospective cohort study. Am J Epidemiol. 2007;166(4):421–8. 10.1093/aje/kwm099 17573337

[94] Chiliza B, Asmal L, Oosthuizen P, van Niekerk E, Erasmus R, Kidd M, et al. Changes in body mass and metabolic profiles in patients with first-episode schizophrenia treated for 12 months with a first-generation antipsychotic. Eur Psychiatry. 2015;30(2):277–83. 10.1016/j.eurpsy.2014.11.013 25577186

[95] Chung S, Popkin BM, Domino ME, Stearns SC. Effect of retirement on eating out and weight change: an analysis of gender differences. Obesity (Silver Spring). 2007;15(4):1053–60. 10.1038/oby.2007.538 17426342

[96] Coutinho T, Bailey KR, Turner ST, Kullo IJ. Arterial stiffness is associated with increase in blood pressure over time in treated hypertensives. J Am Soc Hypertens. 2014;8(6):414–21. 10.1016/j.jash.2014.03.330 PMC4103613 24952654

[97] Crawford D, Ball K, Cleland V, Thornton L, Abbott G, McNaughton SA, et al. Maternal efficacy and sedentary behavior rules predict child obesity resilience. BMC Obes. 2015;2:26. 10.1186/s40608-015-0057-1 PMC4511554 26217541

[98] Criswell R, Lenters V, Mandal S, Stigum H, Iszatt N, Eggesbø M. Persistent environmental toxicants in breast milk and rapid infant growth. Ann Nutr Metab. 2017;70(3):210–6. 10.1159/000463394 28301833

[99] Cummings DM, Henes S, Kolasa KM, Olsson J, Collier D. Insulin resistance status: predicting weight response in overweight children. Arch Pediatr Adolesc Med. 2008;162(8):764–8. 10.1001/archpedi.162.8.764 18678809

[100] Duchin O, Marin C, Mora-Plazas M, Mendes de Leon C, Lee JM, Baylin A, et al. A prospective study of body image dissatisfaction and BMI change in school-age children. Public Health Nutr. 2015;18(2):322–8. 10.1017/S1368980014000366 PMC10270958 24702970

[101] Edwards ER, Hazkani I, Stein E, Josefson JL, Samis JH, Miller JL, et al. Total thyroidectomy and subsequent weight gain in pediatric populations. Laryngoscope. 2023;133(6):1518–23. 10.1002/lary.30532 36537365

[102] Farzan SF, Karagas MR, Jiang J, Wu F, Liu M, Newman JD, et al. Gene-arsenic interaction in longitudinal changes of blood pressure: findings from the Health Effects of Arsenic Longitudinal Study (HEALS) in Bangladesh. Toxicol Appl Pharmacol. 2015;288(1):95–105. 10.1016/j.taap.2015.07.017 PMC4606937 26220686

[103] Fernandes MT, Ferraro AA, Pires A, Santos E, Schvartsman C. Early-life weight and weight gain as predictors of obesity in Brazilian adolescents. Clinics (Sao Paulo). 2013;68(11):1408–12. 10.6061/clinics/2013(11)04 PMC3812563 24270951

[104] Fernandez-Egea E, Miller B, Garcia-Rizo C, Bernardo M, Kirkpatrick B. Metabolic effects of olanzapine in patients with newly diagnosed psychosis. J Clin Psychopharmacol. 2011;31(2):154–9. 10.1097/JCP.0b013e31820fcea3 PMC4390140 21346617

[105] Ferreira S, Mendes J, Couto D, Ferreira D, Rêgo C. Nonalcoholic fatty liver disease and continuous metabolic syndrome in adolescents with overweight/obesity. Acta Med Port. 2024;37(3):177–86. 10.20344/amp.19834 38330918

[106] Fowler SP, Williams K, Hazuda HP. Diet soda intake is associated with long-term increases in waist circumference in a biethnic cohort of older adults: the San Antonio Longitudinal Study of Aging. J Am Geriatr Soc. 2015;63(4):708–15. 10.1111/jgs.13376 PMC4498394 25780952

[107] Funtikova AN, Benítez-Arciniega AA, Gomez SF, Fitó M, Elosua R, Schröder H. Mediterranean diet impact on changes in abdominal fat and 10-year incidence of abdominal obesity in a Spanish population. Br J Nutr. 2014;111(8):1481–7. 10.1017/S0007114513003966 24382017

[108] Geidenstam N, Hsu YH, Astley CM, Mercader JM, Ridderstråle M, Gonzalez ME, et al. Using metabolite profiling to construct and validate a metabolite risk score for predicting future weight gain. PLoS ONE. 2019;14(9):e0222445. 10.1371/journal.pone.0222445 PMC6764659 31560688

[109] Gerards SM, Gubbels JS, Dagnelie PC, Kremers SP, Stafleu A, de Vries NK, et al. Parental perception of child’s weight status and subsequent BMIz change: the KOALA birth cohort study. BMC Public Health. 2014;14:291. 10.1186/1471-2458-14-291 PMC3983903 24678601

[110] Gerber LM, Schwartz JE, Pickering TG. Albumin-to-creatinine ratio predicts change in ambulatory blood pressure in normotensive persons: a 7.5-year prospective study. Am J Hypertens. 2006;19(2):220–6. 10.1016/j.amjhyper.2005.07.025 16448897

[111] Gomes D, von Kries R, Delius M, Mansmann U, Nast M, Stubert M, et al. Late-pregnancy dysglycemia in obese pregnancies after negative testing for gestational diabetes and risk of future childhood overweight: an interim analysis from a longitudinal mother-child cohort study. PLoS Med. 2018;15(10):e1002681. 10.1371/journal.pmed.1002681 PMC6205663 30372451

[112] Gu Y, Scarmeas N, Cosentino S, Brandt J, Albert M, Blacker D, et al. Change in body mass index before and after Alzheimer’s disease onset. Curr Alzheimer Res. 2014;11(4):349–56. 10.2174/1567205010666131120110930 PMC4026350 24251397

[113] Hagman E, Danielsson P, Elimam A, Marcus C. The effect of weight loss and weight gain on blood pressure in children and adolescents with obesity. Int J Obes (Lond). 2019;43(10):1988–94. 10.1038/s41366-019-0384-2 31152153

[114] Hasler G, Lissek S, Ajdacic V, Milos G, Gamma A, Eich D, et al. Major depression predicts an increase in long-term body weight variability in young adults. Obes Res. 2005;13(11):1991–8. 10.1038/oby.2005.244 16339131

[115] Hesketh K, Carlin J, Wake M, Crawford D. Predictors of body mass index change in Australian primary school children. Int J Pediatr Obes. 2009;4(1):45–53. 10.1080/17477160802191122 18608636

[116] Hill C, Saxton J, Webber L, Blundell J, Wardle J. The relative reinforcing value of food predicts weight gain in a longitudinal study of 7--10-y-old children. Am J Clin Nutr. 2009;90(2):276–81. 10.3945/ajcn.2009.27479 19535428

[117] Hollis JL, Crozier SR, Inskip HM, Cooper C, Godfrey KM, Harvey NC, et al. Modifiable risk factors of maternal postpartum weight retention: an analysis of their combined impact and potential opportunities for prevention. Int J Obes (Lond). 2017;41(7):1091–8. 10.1038/ijo.2017.78 PMC5500180 28337028

[118] Hu J, Aris IM, Lin PD, Rifas-Shiman SL, Perng W, Woo Baidal JA, et al. Longitudinal associations of modifiable risk factors in the first 1000 days with weight status and metabolic risk in early adolescence. Am J Clin Nutr. 2021;113(1):113–22. 10.1093/ajcn/nqaa297 PMC7779210 33184628

[119] Huelsing J, Kanafani N, Mao J, White NH. Camp jump start: effects of a residential summer weight-loss camp for older children and adolescents. Pediatrics. 2010;125(4):e884–90. 10.1542/peds.2009-1007 20194273

[120] Inge TH, Jenkins TM, Zeller M, Dolan L, Daniels SR, Garcia VF, et al. Baseline BMI is a strong predictor of nadir BMI after adolescent gastric bypass. J Pediatr. 2010;156(1):103-8.e1. 10.1016/j.jpeds.2009.07.028 PMC2886665 19775700

[121] Ishizaki M, Nakagawa H, Morikawa Y, Honda R, Yamada Y, Kawakami N. Influence of job strain on changes in body mass index and waist circumference–6-year longitudinal study. Scand J Work Environ Health. 2008;34(4):288–96. 18815715

[122] Ix JH, Wassel CL, Chertow GM, Koster A, Johnson KC, Tylavsky FA, et al. Fetuin-A and change in body composition in older persons. J Clin Endocrinol Metab. 2009;94(11):4492–8. 10.1210/jc.2009-0916 PMC2775641 19820014

[123] Jansen H, Wijga AH, Scholtens S, Koppelman GH, Postma DS, Brunekreef B, et al. Change in HbA1c levels between the age of 8 years and the age of 12 years in Dutch children without diabetes: the PIAMA birth cohort study. PLoS ONE. 2015;10(4):e0119615. 10.1371/journal.pone.0119615 PMC4395421 25875773

[124] Johnson MS, Figueroa-Colon R, Herd SL, Fields DA, Sun M, Hunter GR, et al. Aerobic fitness, not energy expenditure, influences subsequent increase in adiposity in black and white children. Pediatrics. 2000;106(4):E50. 10.1542/peds.106.4.e50 11015545

[125] Jourdan C, Brugel D, Hubeaux K, Toure H, Laurent-Vannier A, Chevignard M. Weight gain after childhood traumatic brain injury: a matter of concern. Dev Med Child Neurol. 2012;54(7):624–8. 10.1111/j.1469-8749.2012.04291.x 22524689

[126] Kagura J, Feeley AB, Micklesfield LK, Pettifor JM, Norris SA. Association between infant nutrition and anthropometry, and pre-pubertal body composition in urban South African children. J Dev Orig Health Dis. 2012;3(6):415–23. 10.1017/S2040174412000475 25084294

[127] Karaolis-Danckert N, Buyken AE, Bolzenius K, Perim de Faria C, Lentze MJ, Kroke A. Rapid growth among term children whose birth weight was appropriate for gestational age has a longer lasting effect on body fat percentage than on body mass index. Am J Clin Nutr. 2006;84(6):1449–55. 10.1093/ajcn/84.6.1449 17158429

[128] Katz-Wise SL, Jun HJ, Corliss HL, Jackson B, Haines J, Austin SB. Child abuse as a predictor of gendered sexual orientation disparities in body mass index trajectories among U.S. youth from the Growing Up Today Study. J Adolesc Health. 2014;54(6):730–8. 10.1016/j.jadohealth.2013.11.006 PMC4035383 24388110

[129] Kimokoti RW, Newby PK, Gona P, Zhu L, Jasuja GK, Pencina MJ, et al. Diet quality, physical activity, smoking status, and weight fluctuation are associated with weight change in women and men. J Nutr. 2010;140(7):1287–93. 10.3945/jn.109.120808 PMC2884329 20484553

[130] Koolhaas CM, Kocevska D, Te Lindert BHW, Erler NS, Franco OH, Luik AI, et al. Objectively measured sleep and body mass index: a prospective bidirectional study in middle-aged and older adults. Sleep Med. 2019;57:43–50. 10.1016/j.sleep.2019.01.034 30897455

[131] Kulendran M, Borovoi L, Purkayastha S, Darzi A, Vlaev I. Impulsivity predicts weight loss after obesity surgery. Surg Obes Relat Dis. 2017;13(6):1033–40. 10.1016/j.soard.2016.12.031 28258827

[132] Lange HL, Belury MA, Secic M, Thomas A, Bonny AE. Dietary intake and weight gain among adolescents on depot medroxyprogesterone acetate. J Pediatr Adolesc Gynecol. 2015;28(3):139–43. 10.1016/j.jpag.2014.04.004 PMC4457940 26046602

[133] Lee JJ, Pedley A, Hoffmann U, Massaro JM, Fox CS. Association of changes in abdominal fat quantity and quality with incident cardiovascular disease risk factors. J Am Coll Cardiol. 2016;68(14):1509–21. 10.1016/j.jacc.2016.06.067 PMC5599249 27687192

[134] Licht CM, de Geus EJ, Penninx BW. Dysregulation of the autonomic nervous system predicts the development of the metabolic syndrome. J Clin Endocrinol Metab. 2013;98(6):2484–93. 10.1210/jc.2012-3104 23553857

[135] Lowe MR, Feig EH, Winter SR, Stice E. Short-term variability in body weight predicts long-term weight gain. Am J Clin Nutr. 2015;102(5):995–9. 10.3945/ajcn.115.115402 PMC4625595 26354535

[136] Lyratzopoulos G, Heller RF, Hanily M, Lewis PS. Deprivation status and mid-term change in blood pressure, total cholesterol and smoking status in middle life: a cohort study. Eur J Cardiovasc Prev Rehabil. 2007;14(6):844–50. 10.1097/HJR.0b013e3282efb858 18043309

[137] Machado AV, Camelo LV, Menezes ST, Guimarães JMN, Chor D, Griep RH, et al. Racial discrimination predicts the increase in body weight and BMI in Black individuals from ELSA-Brasil cohort. Cien Saude Colet. 2023;28(6):1655–62. 10.1590/1413-81232023286.14992022 37255143

[138] Martin A, Scahill L, Anderson GM, Aman M, Arnold LE, McCracken J, et al. Weight and leptin changes among risperidone-treated youths with autism: 6-month prospective data. Am J Psychiatry. 2004;161(6):1125–7. 10.1176/appi.ajp.161.6.1125 15169706

[139] Martin K, Radler DR, Sackey J, Zhang C, Shrestha K, Shrestha A, et al. Association between 1(st) trimester diet quality & gestational weight gain rate among pregnant women in Dhulikhel. Nepal BMC Nutr. 2022;8(1):129. 10.1186/s40795-022-00623-7 PMC9650875 36369060

[140] Matsuo T, Murotake Y, Kim MJ, Akiba T, Shimojo N, Kim MK, et al. High general self-efficacy is associated with less weight loss under a supervised dietary modification program. Obes Res Clin Pract. 2010;4(2):e83–162. 10.1016/j.orcp.2009.12.002 24345652

[141] Matsushita Y, Nakagawa T, Yamamoto S, Takahashi Y, Yokoyama T, Mizoue T, et al. Effect of longitudinal changes in visceral fat area and other anthropometric indices to the changes in metabolic risk factors in Japanese men: the Hitachi Health Study. Diabetes Care. 2012;35(5):1139–43. 10.2337/dc11-1320 PMC3329817 22432120

[142] Matthews KA, Crawford SL, Chae CU, Everson-Rose SA, Sowers MF, Sternfeld B, et al. Are changes in cardiovascular disease risk factors in midlife women due to chronological aging or to the menopausal transition. J Am Coll Cardiol. 2009;54(25):2366–73. 10.1016/j.jacc.2009.10.009 PMC2856606 20082925

[143] Monthé-Drèze C, Aris IM, Rifas-Shiman SL, Shivappa N, Hebert JR, Oken E, et al. The role of prenatal psychosocial stress in the associations of a proinflammatory diet in pregnancy with child adiposity and growth trajectories. JAMA Netw Open. 2023;6(1):e2251367. 10.1001/jamanetworkopen.2022.51367 PMC9860526 36662527

[144] Mosquera PS, Villamor E, Malta MB, Cardoso MA. Gestational weight gain and breastfeeding practices in relation to offspring body mass index among Amazonian young children. Am J Hum Biol. 2023;35(2):e23824. 10.1002/ajhb.23824 36301153

[145] Murer SB, Knöpfli BH, Aeberli I, Jung A, Wildhaber J, Wildhaber-Brooks J, et al. Baseline leptin and leptin reduction predict improvements in metabolic variables and long-term fat loss in obese children and adolescents: a prospective study of an inpatient weight-loss program. Am J Clin Nutr. 2011;93(4):695–702. 10.3945/ajcn.110.002212 21289223

[146] Mwamburi DM, Wilson IB, Jacobson DL, Spiegelman D, Gorbach SL, Knox TA, et al. Understanding the role of HIV load in determining weight change in the era of highly active antiretroviral therapy. Clin Infect Dis. 2005;40(1):167–73. 10.1086/426591 15614708

[147] Nouwen A, Ford T, Balan AT, Twisk J, Ruggiero L, White D. Longitudinal motivational predictors of dietary self-care and diabetes control in adults with newly diagnosed type 2 diabetes mellitus. Health Psychol. 2011;30(6):771–9. 10.1037/a0024500 21707174

[148] Nüesch R, Wang Q, Elzi L, Bernasconi E, Weber R, Cavassini M, et al. Risk of cardiovascular events and blood pressure control in hypertensive HIV-infected patients: Swiss HIV Cohort Study (SHCS). J Acquir Immune Defic Syndr. 2013;62(4):396–404. 10.1097/QAI.0b013e3182847cd0 23288033

[149] O’Neill KN, Bell JA, Davey Smith G, Tilling K, Kearney PM, O’Keeffe LM. Puberty timing and sex-specific trajectories of systolic blood pressure: a prospective cohort study. Hypertension. 2022;79(8):1755–64. 10.1161/HYPERTENSIONAHA.121.18531 PMC9278704 35587023

[150] Ohlsson T, Nilsson PM, Persson M, Melander O. Midregional proadrenomedullin predicts reduced blood pressure and glucose elevation over time despite enhanced progression of obesity markers. J Hypertens. 2019;37(3):590–5. 10.1097/HJH.0000000000001893 30540625

[151] Olson KL, Lillis J, Graham Thomas J, Wing RR. Prospective evaluation of internalized weight bias and weight change among successful weight-loss maintainers. Obesity (Silver Spring). 2018;26(12):1888–92. 10.1002/oby.22283 PMC6249105 30421843

[152] Petrie JR, Malik MO, Balkau B, Perry CG, Højlund K, Pataky Z, et al. Euglycemic clamp insulin sensitivity and longitudinal systolic blood pressure: role of sex. Hypertension. 2013;62(2):404–9. 10.1161/HYPERTENSIONAHA.111.00439 23734006

[153] Plumb LA, Pitcher D, Tse Y, Shield JP, Inward C, Sinha MD. Longitudinal changes in body mass index following renal transplantation in UK children. Nephrol Dial Transplant. 2014;29(1):196–203. 10.1093/ndt/gft395 24081859

[154] Rosenbaum K, Wang J, Pierson RN Jr., Kotler DP. Time-dependent variation in weight and body composition in healthy adults. JPEN J Parenter Enteral Nutr. 2000;24(2):52–5. 10.1177/014860710002400252 10772182

[155] Saland JM, Kupferman JC, Pierce CB, Flynn JT, Mitsnefes MM, Warady BA, et al. Change in dyslipidemia with declining glomerular filtration rate and increasing proteinuria in children with CKD. Clin J Am Soc Nephrol. 2019;14(12):1711–8. 10.2215/CJN.03110319 PMC6895497 31712386

[156] Schubert CM, Rogers NL, Remsberg KE, Sun SS, Chumlea WC, Demerath EW, et al. Lipids, lipoproteins, lifestyle, adiposity and fat-free mass during middle age: the Fels Longitudinal Study. Int J Obes (Lond). 2006;30(2):251–60. 10.1038/sj.ijo.0803129 16247511

[157] Sob C, Siegrist M, Hagmann D, Hartmann C. A longitudinal study examining the influence of diet-related compensatory behavior on healthy weight management. Appetite. 2021;156:104975. 10.1016/j.appet.2020.104975 32966848

[158] Sockalingam S, Hawa R, Wnuk S, Santiago V, Kowgier M, Jackson T, et al. Psychosocial predictors of quality of life and weight loss two years after bariatric surgery: results from the Toronto Bari-PSYCH study. Gen Hosp Psychiatry. 2017;47:7–13. 10.1016/j.genhosppsych.2017.04.005 28807141

[159] Starr JM, Deary IJ. Blood pressure, socio-economic status and health in the Lothian 1921 birth cohort: a longitudinal study. Public Health. 1921;125(4):196–200. 10.1016/j.puhe.2010.11.017 21420696

[160] Stice E, Durant S. Elevated objectively measured but not self-reported energy intake predicts future weight gain in adolescents. Appetite. 2014;81:84–8. 10.1016/j.appet.2014.06.012 PMC4128488 24930597

[161] Su S, Wang X, Pollock JS, Treiber FA, Xu X, Snieder H, et al. Adverse childhood experiences and blood pressure trajectories from childhood to young adulthood: the Georgia Stress and Heart Study. Circulation. 2015;131(19):1674–81. 10.1161/CIRCULATIONAHA.114.013104 PMC4430378 25858196

[162] Tanofsky-Kraff M, Yanovski SZ, Schvey NA, Olsen CH, Gustafson J, Yanovski JA. A prospective study of loss of control eating for body weight gain in children at high risk for adult obesity. Int J Eat Disord. 2009;42(1):26–30. 10.1002/eat.20580 PMC2673525 18720473

[163] Taylor BS, Liang Y, Garduño LS, Walter EA, Gerardi MB, Anstead GM, et al. High risk of obesity and weight gain for HIV-infected uninsured minorities. J Acquir Immune Defic Syndr. 2014;65(2):e33-40. 10.1097/QAI.0000000000000010 PMC3957274 24121754

[164] Taylor RW, Williams SM, Galland BC, Farmer VL, Meredith-Jones KA, Schofield G, et al. Quantity versus quality of objectively measured sleep in relation to body mass index in children: cross-sectional and longitudinal analyses. Int J Obes (Lond). 2020;44(4):803–11. 10.1038/s41366-020-0552-4 32099105

[165] Tomiyama AJ, Puterman E, Epel ES, Rehkopf DH, Laraia BA. Chronic psychological stress and racial disparities in body mass index change between Black and White girls aged 10-19. Ann Behav Med. 2013;45(1):3–12. 10.1007/s12160-012-9398-x PMC11412232 22993022

[166] Torres JM, Deardorff J, Holland N, Harley KG, Kogut K, Long K, et al. Deportation worry, cardiovascular disease risk factor trajectories, and incident hypertension: a community-based cohort study. J Am Heart Assoc. 2019;8(23):e013086. 10.1161/JAHA.119.013086 PMC6912982 31771437

[167] Torres L, Lee JL, Park S, Di Lorenzo RC, Branam JP, Fraser SA, et al. Retention, fasting patterns, and weight loss with an intermittent fasting app: large-scale, 52-week observational study. JMIR Mhealth Uhealth. 2022;10(10):e35896. 10.2196/35896 PMC9579929 36194463

[168] van der Valk ES, van der Voorn B, Iyer AM, Mohseni M, Leenen PJM, Dik WA, et al. Hair cortisol, obesity and the immune system: results from a 3 year longitudinal study. Psychoneuroendocrinology. 2021;134:105422. 10.1016/j.psyneuen.2021.105422 34666286

[169] Vis M, Nurmohamed MT, Wolbink G, Voskuyl AE, de Koning M, van de Stadt R, et al. Short term effects of infliximab on the lipid profile in patients with rheumatoid arthritis. J Rheumatol. 2005;32(2):252–5. 15693084

[170] Wang Z, Wang W, Gong R, Yao H, Fan M, Zeng J, et al. Eradication of Helicobacter pylori alleviates lipid metabolism deterioration: a large-cohort propensity score-matched analysis. Lipids Health Dis. 2022;21(1):34. 10.1186/s12944-022-01639-5 PMC8978376 35369887

[171] Wiechers C, Doll JN, Maas C, Gründler K, Büchner K, Poets CF, et al. Enteral feeding advancement and growth until 5 years in extremely preterm infants. BMC Pediatr. 2021;21(1):420. 10.1186/s12887-021-02878-8 PMC8459503 34556084

[172] Williams RV, Zak V, Ravishankar C, Altmann K, Anderson J, Atz AM, et al. Factors affecting growth in infants with single ventricle physiology: a report from the Pediatric Heart Network Infant Single Ventricle Trial. J Pediatr. 2011;159(6):1017-22.e2. 10.1016/j.jpeds.2011.05.051 PMC3215805 21784436

[173] Wren GM, Koutoukidis DA, Scragg J, Whitman M, Jebb S. The association between goal setting and weight loss: prospective analysis of a community weight loss program. J Med Internet Res. 2023;25:e43869. 10.2196/43869 PMC10357317 37405833

[174] Xiao H, Ding K, Li X, Zhou Z, Ma Y, Dai X, et al. Long-term sleep irregularity is associated with elevated cumulative blood pressure in older adults: evidence from a mobile health five-year longitudinal study. Sleep Med. 2025;128:196–205. 10.1016/j.sleep.2025.01.034 39970698

[175] Xin Y, Hertle E, van der Kallen CJH, Schalkwijk CG, Stehouwer CDA, van Greevenbroek MMJ. Longitudinal associations of the alternative and terminal pathways of complement activation with adiposity: the CODAM study. Obes Res Clin Pract. 2018;12(3):286–92. 10.1016/j.orcp.2017.11.002 29174517

[176] Yamazaki A, Kinose D, Kawashima S, Tsunoda Y, Matsuo Y, Uchida Y, et al. Predictors of longitudinal changes in body weight, muscle and fat in patients with and ever-smokers at risk of COPD. Respirology. 2023;28(9):851–9. 10.1111/resp.14537 37364930

[177] Ye C, Foster G, Kaczorowski J, Chambers LW, Angeles R, Marzanek-Lefebvre F, et al. The impact of a cardiovascular health awareness program (CHAP) on reducing blood pressure: a prospective cohort study. BMC Public Health. 2013;13:1230. 10.1186/1471-2458-13-1230 PMC3883556 24369050

[178] Zalbahar N, Najman J, McIntyre HD, Mamun A. Parental pre-pregnancy obesity and the risk of offspring weight and body mass index change from childhood to adulthood. Clin Obes. 2017;7(4):206–15. 10.1111/cob.12200 28557382

[179] McIsaac M, Kaban G, Clay A, Berry W, Prasad B. Long-term impact of bariatric surgery on renal outcomes at a community-based publicly funded bariatric program: the Regina Bariatric Study. Can J Kidney Health Dis. 2019;6:2054358119884903. 10.1177/2054358119884903 PMC6820179 31695923

[180] Reeske A, Spallek J, Bammann K, Eiben G, De Henauw S, Kourides Y, et al. Migrant background and weight gain in early infancy: results from the German study sample of the IDEFICS study. PLoS ONE. 2013;8(4):e60648. 10.1371/journal.pone.0060648 PMC3617202 23593270

[181] Reinehr T, Kleber M, Toschke AM. Former small for gestational age (SGA) status is associated to changes of insulin resistance in obese children during weight loss. Pediatr Diabetes. 2010;11(6):431–7. 10.1111/j.1399-5448.2009.00624.x 20051022

[182] Ahmad Kiadaliri A, Clarke PM, Gerdtham UG, Nilsson P, Eliasson B, Gudbjörnsdottir S, et al. Predicting changes in cardiovascular risk factors in type 2 diabetes in the post-UKPDS era: longitudinal analysis of the Swedish National Diabetes Register. J Diabetes Res. 2013;2013:241347. 10.1155/2013/241347 PMC3647571 23671860

[183] Bouman EJ, Beulens JWJ, den Braver NR, Blom MT, Remmelzwaal S, Elders PJM, et al. Social jet lag and (changes in) glycemic and metabolic control in people with type 2 diabetes. Obesity (Silver Spring). 2023;31(4):945–54. 10.1002/oby.23730 36855048

[184] Claudel SE, Adu-Brimpong J, Banks A, Ayers C, Albert MA, Das SR, et al. Association between neighborhood-level socioeconomic deprivation and incident hypertension: a longitudinal analysis of data from the Dallas heart study. Am Heart J. 2018;204:109–18. 10.1016/j.ahj.2018.07.005 PMC6217793 30092412

[185] Woestenenk JW, Dalmeijer GW, van der Ent CK, Houwen RH. The relationship between energy intake and body-growth in children with cystic fibrosis. Clin Nutr. 2019;38(2):920–5. 10.1016/j.clnu.2018.02.005 29472121

[186] Abohashem S, Hassan I, Wasfy JH, Taub PR. Trends and prevalence of the metabolic syndrome among US adults. JAMA. 2026;335(3):274–7. 10.1001/jama.2025.21712 PMC12699394 41379435

[187] Brauer M, Roth GA, Aravkin AY, Zheng P, Abate KH, Abate YH, et al. Global burden and strength of evidence for 88 risk factors in 204 countries and 811 subnational locations, 1990–2021: a systematic analysis for the Global Burden of Disease Study 2021. Lancet. 1990;403(10440):2162–203. 10.1016/S0140-6736(24)00933-4 PMC11120204 38762324

