# Supplementary material for: Statistical methods for analyzing change over time of continuous outcomes in observational health studies: a scoping review of suggested approaches
Source: Syst Rev. 2026 Oct 17;15:273. doi: 10.1186/s13643-026-03361-0 (PMC13675985; doi:10.1186/s13643-026-03361-0)
Supplement: Supplementary file 1 [file 13643_2026_3361_MOESM1_ESM.docx]

**Additional file**

**Statistical methods for analyzing change over time of continuous outcomes in observational health studies: A systematic scoping review of suggested approaches**

Martina Bouka, Eirini Pagkalidou, Solange Gonzalez Chiappe, Tracy Bonsu Osei, Ulrike Grittner, Katharina Nimptsch, Tobias Pischon*^1,2,3^

*Corresponding author: Tobias Pischon ([tobias.pischon@mdc-berlin.de](mailto:tobias.pischon@mdc-berlin.de))

^1^Molecular Epidemiology Research Group, Max Delbrück Center for Molecular Medicine in the Helmholtz Association (MDC), Berlin, Germany; ^2^Biobank Technology Platform, Max Delbrück Center for Molecular Medicine in the Helmholtz Association (MDC), Berlin, Germany; ^3^Charité – University Medical Center Berlin, corporate member of Freie Universität Berlin and Humboldt-Universität zu Berlin, Berlin, Germany

**Contents**

1. **URL to search strategies**
2. **Supplementary Figure**
3. **Supplementary Tables**
1. **URL to search strategies**

*Search strategy for applied studies:*

| 1. | "score"[Title/Abstract] OR "over time"[Title/Abstract] OR "repeated measures"[Title/Abstract] |
| --- | --- |
| 2. | "change"[Title/Abstract] OR "gain"[Title/Abstract] OR "increase"[Title/Abstract] OR "decrease"[Title/Abstract] |
| 3. | "weight"[Title/Abstract] OR "HDL-C"[Title/Abstract] OR "HDL"[Title/Abstract] OR "HbA1c"[Title/Abstract] OR "Triglycerides"[Title/Abstract] OR "Waist Circumference"[Title/Abstract] OR "Blood pressure"[Title/Abstract] |
| 4. | "Cohort "OR "Prospective " OR "Longitudinal" |
| 5. | "predictor"[Title/Abstract] OR "risk factor"[Title/Abstract] |
| 6. | ("2000/01/01"[Date - Publication] : "3000"[Date - Publication]) |
| 7. | #1 AND #2 AND #3 AND #4 AND #5 AND #6 |

*Search strategy for methodological studies:*

| 1. | "change"[Title/Abstract] OR "change score"[Title/Abstract] OR "change scores"[Title/Abstract] OR "prospective"[Title/Abstract] OR "longitudinal "[Title/Abstract] OR "repeated measures"[Title/Abstract] |
| --- | --- |
| 2. | "baseline adjustment"[Title/Abstract] OR "adjusting for baseline"[Title/Abstract] OR "baseline-adjusted"[Title/Abstract] OR "adjustment for baseline"[Title/Abstract] OR "baseline value"[Title/Abstract] |
| 3. | "regression to the mean"[Title/Abstract] OR "regression toward the mean"[Title/Abstract] OR "regression towards the mean"[Title/Abstract] OR "RTM"[Title/Abstract] OR "measurement error"[Title/Abstract] OR "bias correction"[Title/Abstract] OR "Oldham"[Title/Abstract] OR "Blomqvist"[Title/Abstract] OR "bias adjustment"[Title/Abstract] OR "spurious association"[Title/Abstract] |
| 4. | "Cohort" OR "Observational" OR "epidemiology" OR "Simulation" |
| 5. | ("2000/01/01"[Date - Publication] : "3000"[Date - Publication]) |
| 6. | ((#1 AND #3) OR (#2 AND #3)) AND #4 AND #5 |

2. **Supplementary Figure**

**Figure S1:** Applied studies included in the review by publication year.

3. **Supplementary Tables**

**Table S1:** Methodological approaches proposed to address regression to the mean (RTM) summarized by classes.

| **Regression to the mean (RTM)** | | | | | | |
| --- | --- | --- | --- | --- | --- | --- |
| **Class** | **Research question and answer** | **Studies** | **Approaches included in each class** | **Strengths** | **Limitations** | **Recommendations** |
| **1: Analytical RTM correction formulas** | Need to know how much change to expect purely because of RTM?  *Estimate expected RTM and compare observed vs expected change:* | •**Tannenbaum** 2004 [71]  (applied example)  •**Cameron** 2005 [28]  (methodological/conceptual)  •**Veldhuizen** 2021 [74]  (applied example) | **Tannenbaum**:   - Correlation-based shrinkage formulas that adjust an observed value toward the population mean   **Cameron**:   - Generalized versions allowing different standard deviations between repeated measurements   **Veldhuizen**:   - Subgroup-level expected RTM correction following selection on extreme values | **Tannenbaum**:   - Not reported (formula applied to study data)   **Cameron**:   - Adjustment for RTM separates true catch-up from statistical artifact - Quantitative, reproducible definition of catchup growth - Applicable at group and individual levels   **Veldhuizen**:   - Not reported (formula applied to study data) | **Tannenbaum**:   - Not reported (formula applied to study data)   **Cameron**:   - Assumes stable correlation between z-scores - Individual variability may not be fully captured   **Veldhuizen**:   - Not reported (formula applied to study data) | **Tannenbaum:**   - Not reported (formula applied to study data)   **Cameron**:   - Using the z-score approach adjusted for RTM rather than raw values   **Veldhuizen**:   - Apparent improvement in research may often be produced by containing ME. |
|  | Need to get RTM adjusted prediction?  *Correct observed change to obtain*  *RTM-adjusted estimates:* | •**Schmidt** 2021 [64]  (applied example)  •**Chapurlat** 2001 [32]  (applied example)  •**Brown** 2021 [26]  (applied example) | **Schmidt**:   - RTM was initially evaluated graphically then correlation-based shrinkage formulas that adjust an observed value toward the population mean   **Chapurlat**:   - Inverse-variance weighted estimation of the underlying true baseline value   **Brown**:   - Generalized versions allowing different standard deviations between repeated measurements | **Schmidt**:   - Not reported (formula applied to study data)   **Chapurlat**:   - Adjustment for RTM allowed more accurate estimation of true changes - Demonstrated practical implications for clinical decision-making.   **Brown**:   - Not reported (formula applied to study data) | **Schmidt**:   - Not reported (formula applied to study data)   **Chapurlat**:   - RTM adjustment relies on assumptions about distribution and variance of measurements - Confidence intervals remained wide, reflecting high variability.   **Brown**:   - Not reported (formula applied to study data) | **Schmidt**:   - Not reported (formula applied to study data)   **Chapurlat**:   - Interpret early extreme changes in bone markers cautiously (they may represent RTM rather than true biological effect) - Use repeated measurements and RTM adjustment to estimate true biological changes in clinical trials.   **Brown**:   - Not reported (formula applied to study data) |
|  | Need to adjust observed change?  *Use regression-adjusted prediction methods that account for RTM when forecasting future values* | •**Cole** 2011 [35]  (methodological/conceptual) | **Cole**:   - Generalized versions allowing different standard deviations between repeated measurements | **Cole**:   - Not reported (formula applied to study data) | **Cole**:   - Not reported (formula applied to study data) | **Cole**:   - When adjusting for RTM, reducing bias in early/late developers. |
|  | Need a more general framework for detecting and correcting RTM?  *Apply general RTM correction and*  *diagnostic methods, including adjusted*  *differences and variance-based tests* | •**Kelly** 2005 [49]  (methodological/conceptual) | **Kelly**:   - Correlation-based shrinkage formulas that adjust an observed value toward the population mean | **Kelly**:   - Provides generalizable framework for detecting and correcting RTM - Applicable across multiple traits, species, and disciplines. | **Kelly**:   - Adjustment methods may be conservative or rely on strong assumptions - Cannot fully correct for regression effect if postulated causal factor is unmeasured. | **Kelly**:   - Always consider RTM when analysing repeated measures or comparing groups with different initial means - Use adjusted differences or variance-based tests to account for regression effect in observational studies - Include control groups when possible to distinguish true causal effects from statistical artifacts. |
| **2: Model-based longitudinal approaches** | Need to estimate individual growth/change trajectories while directly accounting for RTM?  *Use conditional or mixed-effects growth models that incorporate baseline values and repeated measurements* | •**Leung** 2017 [52] (child growth/velocity)  (methodological/conceptual)  •**Ripatti** 2008 [60]  (methodological/conceptual)  •**Gmel** 2008 [41] (panel data and intervention-related change)  (applied example)  •**Chiolero** 2013 [79]  (simulation) | **Leung:**   - Conditional Random Slope (CRS) model - Linear mixed-effects model with baseline × time interaction - Accounts for RTM across repeated measurements   **Ripatti:**   - Conditional modelling framework for panel data with ≥3 measurements - Models the relationship between initial level and subsequent change while controlling for RTM   **Gmel:**   - Oldham’s method to assess baseline–change correlations - Barnett method to quantify and adjust for RTM - Growth-curve modelling to estimate change while accounting for ME and individual differences   **Chiolero:**   - Compared multiple RTM methods under varying ME and between-individual variability - Methods included variance comparison, Oldham’s, repeated-measurement, Blomqvist’s, and linear random-effects regression | **Leung:**   - Generalizes the classical conditional velocity model - Flexibly handles multiple measurements using mixed models - Directly addresses RTM while maintaining interpretability - Demonstrated predictive validity in two independent cohorts   **Ripatti:**   - Flexible regression modelling and standard statistical inference - Allows incorporation of additional variables - Allows testing of the significance and form of deviations from expected patterns - Can be implemented using standard statistical software   **Gmel:**   - Combines multiple methods to detect and adjust for RTM - Accommodates individual differences and multiple measurement points - Models short- and long-term effects - Improves separation of true change from ME   **Chiolero:**   - Directly compares several established methods - Examines multiple simulation scenarios with realistic BMI growth parameters - Explicitly examines ME and variability in change - Provides guidance according to the number of measurement waves and available ME information | **Leung:**   - Limited to two cohorts, reducing generalizability - CRS did not outperform simpler conditional SDS models with balanced data - Further validation needed for other outcomes and data structures   **Ripatti:**   - General limitations of alcohol consumption panel data may affect the validity of empirical results   **Gmel:**   - Hierarchical linear modelling restricted to participants with complete data at all four time points - Imperfect measurement instruments may leave residual RTM - More pre-intervention measurements or more reliable instruments may improve accuracy   **Chiolero:**   - Assumed linear growth and no confounding - Relatively small simulation sample (n = 500) - Simulated distributions may not represent all real-world settings - Linear random-effects models require multiple measurements - No satisfactory method identified for two-wave data when linear growth is inappropriate | **Leung:**   - Use the Conditional Random Slope (CRS) model   **Ripatti:**   - Use conditional models to assess intervention-related changes by initial consumption while accounting for RTM   **Gmel:**   - Account for RTM in both study design and analysis - Use ≥2 baseline measurements where possible - Use growth-curve modelling to estimate true change and subgroup differences - Ensure sufficient measurements to capture short- and long-term effects - Use reliable measurement instruments   **Chiolero:**   - Use multiple measurements whenever possible - With >2 waves, use linear random-effects modelling - With two waves and accurate ME information, consider Blomqvist’s method - Interpret baseline-change associations cautiously, considering ME and variability in change |
|  | Need to distinguish meaningful individual change from RTM or test-retest variability?  *Use reliable-change, Bayesian predictive, or regression-based change methods that compare observed change with expected change* | •**Li Y** 2023 [53]  (simulation)  •**Marsden** 2011 [55]  (applied example)  •**Cysique** 2009 [36]  (simulation) | **Li Y:**   - Bayesian bivariate regression using the posterior predictive distribution - Accounts for RTM and uncertainty in predicted post-test scores - Incorporates prior distributions to identify meaningful change in health-related quality of life   **Marsden:**   - Assessed self-reported substance use before admission and clinical review - Used multilevel mixed-linear modelling with observed and true scores to estimate RTM   **Cysique:**   - Used regression-based change scores derived from a neuropsychologically stable normative sample to correct for RTM - Adjusted for practice effects and test–retest variability using normative regression-based expectations | **Li Y:**   - Accounts for RTM and post-test drift - Incorporates prior information and quantifies uncertainty - Performs well when measurement reliability is low - Allows prior information to be updated as new evidence becomes available   **Marsden:**   - Allows direct assessment of RTM using data from the treatment population   **Cysique:**   - Accounts for RTM, practice effects, and test–retest variability | **Li Y:**   - More computationally complex than traditional methods - Sensitive to biased priors, particularly with small samples - May be more conservative and miss some true changes - Requires careful prior specification and validation   **Marsden:**   - Conservative for assessing improvement   **Cysique:**   - Uses separate regression equations for different intervals - May not fully account for practice effects at weeks 36 and 48 | **Li Y:**   - Consider the Bayesian posterior predictive distribution method when measurement reliability is low or RTM may distort change detection   **Marsden:**   - Use the Jacobson and Truax Reliable Change Index to evaluate change in substance use disorder treatment - Account for RTM when evaluating change   **Cysique:**   - Not reported (method applied to study data) |
|  | Need to model population or subgroup change over time when no randomized control group exists?  *Compare expected RTM-driven patterns with observed effect patterns before interpreting prospective associations as causal* | • **Sorjonen** 2023 [68]  (methodological/conceptual) | **Sorjonen:**   - Derived expected effect patterns under true causal effects versus spurious RTM effects - Computed cross-lagged, reversed, and change-score effects using published correlations - Performed six random-effects meta-analyses of baseline-adjusted and change-based effects - Compared observed effect patterns with those expected from RTM - Simulated 1,000 participants with no true prospective effects to examine RTM-induced spurious associations | **Sorjonen:**   - Not reported (meta-analysis) | **Sorjonen**:   - Not reported (meta-analysis) | **Sorjonen:**   - Rule out alternative explanations before inferring true prospective effects |
|  | Need to evaluate whether prospective/baseline-adjusted effects may be spurious because of RTM?  *Use longitudinal/panel models or growth-curve approaches to estimate true change net of RTM and compare subgroup trajectories* | •**Skog** 2016 [66] (best for when no control group exists)  (methodological/conceptual)  •**Ripatti** 2008 [60]  (methodological/conceptual)  •**Gmel** 2008 [41]  (applied example) | **Skog:**   - Developed a theoretical model using repeated-measures log-linear regression - Demonstrated that flux with a stationary marginal distribution can produce RTM even without ME   **Ripatti:**   - Conditional modelling framework for panel data with ≥3 measurements - Models the relationship between initial consumption and subsequent change while controlling for RTM   **Gmel:**   - Used Oldham’s method to assess baseline–change correlations - Applied the Barnett method to quantify and adjust for RTM - Used hierarchical growth-curve modelling to estimate true change while accounting for ME and individual differences | **Skog:**   - Avoids inappropriate external control comparisons - Applicable when no randomized control group is available - Can be applied by constructing a regression curve without control data   **Ripatti:**   - Flexible regression modelling with standard statistical inference - Allows incorporation of additional variables - Allows testing of the significance and form of deviations from expected patterns - Can be implemented using standard statistical software   **Gmel:**   - Combines multiple methods to detect and adjust for RTM - Accommodates individual differences and multiple measurement points - Models short- and long-term effects - Improves separation of true change from ME | **Skog:**   - A randomized control group would be preferable   **Ripatti:**   - General limitations of alcohol consumption panel data may affect the validity of empirical results   **Gmel:**   - Hierarchical linear modelling restricted to participants with complete data at all four time points - Imperfect measurement instruments may leave residual RTM - More pre-intervention measurements or more reliable instruments may improve accuracy | **Skog:**   - Not reported   **Ripatti:**   - Use conditional models to assess intervention-related changes by initial consumption while controlling for RTM   **Gmel:**   - Account for RTM in both study design and analysis - Use ≥2 baseline measurements where possible - Use growth-curve modelling to estimate true change and subgroup differences - Ensure sufficient measurements to capture short- and long-term effects - Use reliable measurement instruments and multiple baseline measurements for stable subgroup classification |
| **3: Methods addressing baseline-change associations** | Need to choose the correct method for baseline-change association?  *Match the method to research question.* | •**Tu** 2005 [72]  (methodological/conceptual)  •**Tu** 2007 [6]  (methodological/conceptual)  •**Chiolero** 2013 [79]  (simulation) | **Tu 2005:**   - Examined Oldham’s method and Blomqvist’s formula   **Tu 2007:**   - Examined Oldham’s method, Blomqvist’s formula, the variance-ratio test, structural regression, and multilevel modelling   **Chiolero:**   - Compared multiple methods under varying levels of ME and between-individual variability in change - Methods included crude correlation, variance comparison, Oldham’s method, repeated-measurement methods, Blomqvist’s method, and linear random-effects regression | **Tu 2005:**   - Blomqvist’s formula provides an unbiased estimate of change given a baseline value - Oldham’s method tests the appropriate null hypothesis for differential baseline effects - Avoids interpreting a non-zero regression slope as evidence of a differential baseline effect   **Tu 2007:**   - Blomqvist’s formula corrects bias in the regression slope due to error in initial values - Oldham’s method provides an unbiased test of correlation for differential baseline effects - Avoids potentially misleading baseline–change correlations   **Chiolero:**   - Directly compares several established methods - Examines multiple simulation scenarios with realistic BMI growth parameters - Explicitly examines ME and variability in change - Provides guidance according to the number of measurement waves and available ME information | **Tu 2005:**   - Both methods may be biased when individuals are selected based on high initial values   **Tu 2007:**   - A non-zero Blomqvist regression slope does not necessarily indicate a differential baseline effect - Heterogeneous treatment response is not corrected by Blomqvist’s formula - Oldham’s method can detect whether real change has occurred but does not estimate the effect of baseline   **Chiolero:**   - Assumed linear growth and no confounding - Relatively small simulation sample (n = 500) - Simulated distributions may not represent all real-world settings - Linear random-effects models require multiple measurements - No satisfactory method identified for two-wave data when linear growth is inappropriate | **Tu 2005:**   - Select methods for addressing RTM, ME, or mathematical coupling according to the research question   **Tu 2007:**   - Select the appropriate method according to the research question   **Chiolero:**   - Use multiple measurements whenever possible - With >2 waves, use linear random-effects modelling - With two waves and accurate ME information, Blomqvist’s method provides accurate estimates - Interpret baseline-change associations cautiously, considering ME and variability in change |
|  | Need to check whether the apparent association/change is spurious?  *Use alternative model specifications or compare observed patterns with RTM expectations* | •**Sorjonen** 2022 [69]  (applied example)  •**Veldhuizen** 2021 [74]  (applied example)  •**Dugravot** 2009 [38]  (applied example) | **Sorjonen:**   - Compared latent change-score models with and without regression of change on the initial outcome value - Compared models with different susceptibility to RTM - Used simulation to assess the resulting effects   **Veldhuizen:**   - Estimated expected subgroup-level RTM following selection on extreme values   **Dugravot:**   - Used errors-in-variables regression with test–retest reliability to correct baseline-adjusted estimates for ME - Conducted sensitivity analyses assuming higher ME | **Sorjonen:**   - Demonstrates how different analytical specifications can lead to different conclusions   **Veldhuizen:**   - Not reported (formula applied to study data)   **Dugravot:**   - Applies correction for ME - Compares unadjusted and baseline-adjusted methods | **Sorjonen:**   - Measures of maternal and child intelligence were limited by the instruments used and timing of assessment   **Veldhuizen:**   - Not reported (formula applied to study data)   **Dugravot:**   - Two-wave design limits modelling of nonlinear trajectories - Assumes normally distributed ME | **Sorjonen:**   - Analyse observational data using alternative model specifications - Consider predicting change both forward and backward in time   **Veldhuizen:**   - Consider whether apparent improvement may result from ME and RTM   **Dugravot:**   - Interpret baseline-adjusted analyses cautiously because of potential RTM - Use ME-correction methods when baseline variables are measured with error - Collect multiple waves to better model cognitive trajectories |
|  | Need to compare subgroup change by baseline level?  *Combine baseline-change methods with RTM correction and longitudinal modelling* | •**Gmel** 2008 [41]  (applied example) | **Gmel:**   - Oldham’s method to assess baseline–change correlations - Barnett method to quantify and adjust for RTM - Growth-curve modelling to estimate change while accounting for ME and individual differences | **Gmel:**   - Combines multiple methods to detect and adjust for RTM - Accommodates individual differences and multiple measurement points - Models short- and long-term effects - Improves separation of true change from ME | **Gmel:**   - Hierarchical linear modelling restricted to participants with complete data at all four time points - Imperfect measurement instruments may leave residual RTM - More pre-intervention measurements or more reliable instruments may improve accuracy | **Gmel:**   - Account for RTM in both study design and analysis - Use ≥2 baseline measurements where possible - Use growth-curve modelling to estimate true change and subgroup differences - Ensure sufficient measurements to capture short- and long-term effects - Use reliable measurement instruments and multiple baseline measurements for stable subgroup classification |
| 4: Likelihood / distributional RTM estimators | Want advanced distributional RTM estimation?  *Estimate RTM using likelihood-based methods when data are selected using cut offs and standard RTM assumptions (e.g., normality) may not hold* | •**Umair** 2024 [73]  (simulation) | **Umair:**   - Derived RTM formulas for test statistics following a bivariate t-distribution - Used maximum likelihood estimation to quantify RTM following selection based on a baseline criterion or cut-off | **Umair:**   - First method to address RTM under a bivariate t-distribution - Applicable when baseline and follow-up samples differ - Improved accuracy compared with established RTM methods - Practical and accessible to implement | **Umair**:   - Not reported | **Umair**:   - Account for RTM when participants are selected based on a baseline criterion, particularly without a control group - Consider the proposed method as an alternative to the paired t-test for equal or unequal degrees of freedom |
| 5: Design-stage approaches | Still designing the study?  *Use design features that reduce RTM before data collection* | •**Barnett** 2005 [23]  (methodological/conceptual)  •**Zhang** 2003 [77]  (methodological/conceptual) | **Barnett:**   - Estimate the magnitude of RTM - Analyse data using analysis of covariance - Randomly allocate participants to comparison groups - Select participants based on multiple measurements   **Zhang:**   - Intervention studies: use random assignment and a control group - Non-intervention longitudinal studies: use a separate baseline measure from that used for participant selection - Select participants using measure X and assess baseline using an independent measure Z - Estimate follow-up change as Y − Z to reduce selection-related RTM bias | **Barnett:**   - Not reported   **Zhang:**   - A separate baseline measure can control for regression effects in longitudinal designs - Comparing follow-up with this independent baseline can provide an unbiased estimate of true change | **Barnett:**   - Not reported   **Zhang:**   - True scores were known in the simulation but are generally unknown in real-world research | **Barnett:**   - Consider RTM as a potential explanation for observed change in repeated-measures data - Reduce RTM through appropriate study design and statistical methods   **Zhang:**   - Consider the nature and potential effects of RTM when designing and analysing longitudinal studies |
|  | Already have observational data?  *Avoid creating RTM through participant selection* | •**Soer** 2020 [67]  (applied example)  •**Choy** 2024 [34]  (applied example) | **Soer:**   - Used a fixed-effects model comparing healthcare costs 2 years before and 2 years after intervention - Constructed a conservative adjusted sample assuming participants were not selected based on high healthcare costs 2 years before admission   **Choy:**   - Used paired Wilcoxon signed-rank tests to compare pre- and post-ablation premature ventricular contraction burden within individuals | **Soer:**   - Not reported (method applied to study data)   **Choy:**   - Inclusion of all patients, rather than only those with increased premature ventricular contractions, helps account for RTM - Objective measurement using ambulatory monitoring | **Soer:**   - Not reported (method applied to study data)   **Choy:**   - Small sample size (n = 130), limiting power, particularly for subgroup analyses - Short follow-up (median ~3 months) may miss later changes in premature ventricular contraction burden | **Soer:**   - Not reported (method applied to study data)   **Choy:**   - Conduct larger studies with longer follow-up to assess late effects and potential differences by ablation energy type |
| **6:Causal/estimand guidance & baseline-adjustment critique** | Exposure starts before baseline or affects baseline outcome?  *Avoid routine baseline adjustment; consider change-score models* | •**Glymour** 2005 [2]  (methodological/conceptual)  •**Glymour** 2022 [16]  (methodological/conceptual)  •**Kramer** 2016 [50]  (applied example)  •**Dukes** 2025 [7]  (methodological/conceptual) | **Glymour 2005:**   - Used directed acyclic graphs to examine causal relationships - Compared baseline-adjusted and unadjusted approaches   **Glymour 2022:**   - Commentary on Tennant et al. addressing baseline adjustment and change-score analyses   **Kramer:**   - Compared three models:   - Model 1: Change (t2 − t1), unadjusted for baseline   - Model 2: Follow-up outcome (t2), adjusted for baseline (t1)   - Model 3: Change adjusted for baseline (hybrid; equivalent to Model 2)   **Dukes:**   - Compared change-score and baseline-adjusted approaches from a causal-inference perspective - Examined connections between change-score analyses and difference-in-differences - Provided recommendations based on assumptions about the confounding mechanism | **Glymour 2005:**   - Not reported   **Glymour 2022:**   - Not reported   **Kramer:**   - Model 1: Captures the total causal effect; simple and interpretable; consistent across cohorts - Model 2: Adjusts for baseline and accounts for RTM - Model 3: Accounts for baseline; equivalent to Model 2   **Dukes:**   - Not reported | **Glymour 2005:**   - Not reported   **Glymour 2022:**   - Not reported   **Kramer:**   - **Model 1:** Does not adjust for baseline imbalance, although this may be a minor issue when exposure precedes baseline - **Model 2:** May introduce over-adjustment bias when exposure affects baseline and underestimate the causal effect - **Model 3:** Has the same potential over-adjustment bias as Model 2, with no added benefit   **Dukes:**   - Not reported | **Glymour 2005:**   - Avoid routine baseline adjustment when baseline is measured with error, as this may introduce RTM bias - Consider potential ceiling and floor effects, which baseline adjustment may not resolve   **Glymour 2022:**   - When exposure precedes baseline, distinguish between the effect on change and the direct effect on the follow-up outcome - Use directed acyclic graphs to clarify the relevant causal estimand - Recognize that baseline-adjusted estimates may be biased in settings where change-score estimates are unbiased   **Kramer:**   - **Model 1:** Preferred when exposure affects baseline - **Model 2:** Use when exposure starts at or after baseline; avoid when exposure affects baseline - **Model 3:** Same recommendation as Model 2   **Dukes:**   - Recommendations depend on assumptions about the underlying confounding mechanism |
|  | Exposure starts after baseline or baseline outcome determines exposure?  *Baseline adjustment may be appropriate* | •**Lepage** 2015 [51]  (methodological/conceptual)  •**Gustavson** 2014 [43]  (simulation)  •**Tennant** 2022 [5]  (methodological/conceptual)    •**Dukes** 2025 [7]  (methodological/conceptual)  •**Jones** 2016 [48]  (applied example) | **Lepage:**   - Used directed acyclic graphs and path analysis to identify causal pathways, ME, and confounding - Used simulations to assess bias and standard errors under different scenarios   **Gustavson:**   - Used Monte Carlo simulations to examine different forms of selective attrition and RTM - Distinguished between transient and enduring causes of RTM - Compared multiple regression and change-score methods under different modelling assumptions   **Tennant:**   - Simulated three scenarios in which the baseline outcome was a competing exposure, confounder, or mediator - Compared change-score analyses with appropriate estimators of total and/or direct causal effects   **Dukes:**   - Compared change-score and baseline-adjusted approaches from a causal-inference perspective - Examined connections between change-score analyses and difference-in-differences - Considered method choice according to assumptions about the confounding mechanism   **Jones:**   - Used correlation and linear regression to assess baseline-response relationships - Compared baseline with pre-baseline glycated haemoglobin measurements taken 1-26 weeks earlier - Examined pre-baseline to baseline change to estimate RTM - Applied baseline adjustment and a bias-correction method using repeated baseline measurements to account for ME | **Lepage:**   - Directed acyclic graphs and path analysis clarify causal assumptions - Simulations support theoretical predictions - Identifies conditions in which baseline adjustment may reduce or introduce bias - Applicable to randomized and observational studies   **Gustavson:**   - Multiple regression and change-score analyses were generally robust to selective attrition - Monte Carlo simulations allowed evaluation across multiple scenarios - Compares the performance of multiple regression and change-score analyses - Highlights the importance of modern missing-data methods   **Tennant:**   - Not reported   **Dukes:**   - Not reported   **Jones:**   - Large prospective observational cohorts - Findings assessed across two independent cohorts and two therapies - Repeated pre-baseline glycated haemoglobin measurements allowed assessment of ME and RTM - Bias correction supported the appropriateness of baseline adjustment | **Lepage:**   - Relies on assumptions about unmeasured confounding and causal structure - Does not explore all possible causal structures - Assumes linear relationships and normally distributed outcomes - ME may still bias estimates - Limited generalizability to nonlinear and time-to-event models   **Gustavson:**   - Simulated only selected scenarios and a limited range of predictor–change associations and stability levels - True population values were known in simulations but are unknown in real-world settings - Assumes correct knowledge of whether RTM is present - Use of listwise deletion may not reflect other missing-data approaches   **Tennant:**   - Simulations were simplified and relied on assumptions that may not reflect real-world settings - Potential for multiple confounders of the relationship between waist circumference and insulin concentration   **Dukes:**   - Not reported   **Jones:**   - Clinical treatment decisions may exaggerate RTM | **Lepage:**   - When baseline determines exposure, adjust for baseline - When exposure starts before baseline, prefer an unadjusted model - In nonrandomized studies with potential unmeasured confounding, evaluate the causal structure using directed acyclic graphs - Base the choice of adjustment strategy on the assumed causal structure   **Gustavson:**   - Use multiple regression when RTM is expected because of transient factors - Use change-score analysis when RTM is not expected because of enduring factors - Apply modern missing-data methods, such as multiple imputation - Perform sensitivity analyses when the presence of RTM is uncertain   **Tennant:**   - For causal-effect estimation in observational studies, avoid change-score analyses and consider alternative analytical approaches   **Dukes:**   - Recommendations depend on assumptions about the underlying confounding mechanism   **Jones:**   - Adjust for baseline glycated haemoglobin when analysing predictors of treatment response - Standard baseline regression adjustment is appropriate when repeated baseline measurements are unavailable - Bias correction using repeated baseline measurements supports baseline adjustment - Expressing change as a percentage of baseline does not sufficiently account for baseline confounding |
|  | Unsure whether change-score or baseline-adjusted model is valid?  *Use DAGs/causal assumptions to define the estimand first* | •**Dukes** 2025 [7]  (methodological/conceptual)  •**Glymour** 2022 [16]  (methodological/conceptual)  •**Tennant** 2022 [5]  (methodological/conceptual) | **Dukes:**   - Compared change-score and baseline-adjusted approaches from a causal-inference perspective - Examined connections between change-score analyses and difference-in-differences - Considered method choice according to assumptions about the confounding mechanism   **Glymour 2022:**   - Commentary on Tennant et al. addressing baseline adjustment and change-score analyses   **Tennant:**   - Simulated three scenarios in which the baseline outcome was a competing exposure, confounder, or mediator - Compared change-score analyses with appropriate estimators of total and/or direct causal effects | **Dukes**:   - Not reported   **Glymour 2022:**   - Not reported   **Tennant**:   - Not reported | **Dukes:**   - Not reported   **Glymour 2022:**   - Not reported   **Tennant:**   - Simulations were simplified and relied on distributional assumptions that may not reflect real-world settings - Potential for multiple confounders of the relationship between waist circumference and insulin concentration | **Dukes:**   - Recommendations depend on assumptions about the underlying confounding mechanism   **Glymour 2022:**   - Avoid routine baseline adjustment when baseline is measured with error, as this may introduce RTM bias - Consider potential ceiling and floor effects, which baseline adjustment may not resolve - When exposure precedes baseline, distinguish between the effect on change and the direct effect on the follow-up outcome - Use directed acyclic graphs to clarify the relevant causal estimand - Recognize that baseline-adjusted estimates may be biased in settings where change-score estimates are unbiased   **Tennant:**   - For causal-effect estimation in observational studies, avoid change-score analyses and consider alternative analytical approaches |
|  | Difference-in-differences study and matching on pre-period outcomes?  *Matching on baseline/preperiod values can introduce RTM* | •**Daw** 2018 [37]  (simulation) | **Daw:**   - Quantified bias as the mean absolute deviation of the estimated treatment effect from the true effect across 1,000 simulations - Compared matching on pre-period outcomes, covariates, and pre-period trends - Assessed how different matching strategies introduced or reduced bias | **Daw:**   - Clearly demonstrates RTM bias in difference-in-differences designs with matching - Known true treatment effect allows precise quantification of bias - Tests multiple causal and correlation scenarios - Provides insights for the design and analysis of observational studies | **Daw:**   - Based entirely on simulated data - Assumed normal distributions and first-order autoregressive correlation structures - Examined only a null intervention (true effect = 0) - Considered simplified causal scenarios that may not reflect real-world confounding and correlation patterns | **Daw:**   - Consider potential RTM when constructing matched samples for difference-in-differences analyses - Use caution when matching on baseline outcomes or covariates, as this may introduce RTM bias - When possible, use unmatched samples or consider matching on pre-period trends when serial correlation is high |
|  | Concerned about attrition or missing data together with RTM?  Use sensitivity analyses and modern missing-data methods | •**Gustavson** 2013 [43]  (simulation) | **Gustavson:**   - Used Monte Carlo simulations to examine different forms of selective attrition and RTM - Distinguished between transient and enduring causes of RTM - Compared multiple regression and change-score methods under different modelling assumptions | **Gustavson:**   - Multiple regression and change-score analyses were generally robust to selective attrition - Monte Carlo simulations allowed evaluation across multiple scenarios - Compares multiple regression and change-score analyses - Highlights the importance of modern missing-data methods | **Gustavson:**   - Simulated only selected scenarios, with a limited range of predictor–change associations and stability levels - True population values were known in simulations but are unknown in real-world settings - Assumes correct knowledge of whether RTM is present - Use of listwise deletion may not reflect other missing-data approaches | **Gustavson:**   - Use multiple regression when RTM is expected due to transient factors - Use change-score analysis when RTM is not expected due to enduring factors - Apply modern missing-data methods, such as multiple imputation - Perform sensitivity analyses when the presence of RTM is uncertain - Carefully account for RTM to avoid introducing opposing biases |

^a^Reference numbers for all publications refer to the main text references

^b^List of acronyms: BMI = body mass index; CRS = Conditional Random Slope; ME = measurement error; RTM = regression to the mean; SDS = standard deviation score.

**Table S2:** Methodological approaches proposed to address Measurement Error (ME) summarized by classes

| **ME (ME)** | | | | | | |
| --- | --- | --- | --- | --- | --- | --- |
| **Class** | **Research question and answer** | **Studies** | **Approaches included in each class** | **Strengths** | **Limitations** | **Recommendations** |
| **1: Calibration and validation–based correction** | Device or measurement method changed over time?  *Use calibration methods for longitudinal device differences* | •**Loop** 2025 [54]  (simulation) | **Loop:**   - Compared regression calibration and multiple imputation when the exposure-measurement device changed over time - Assessed bias, standard-error accuracy, and efficiency - Examined varying sample sizes, calibration-study proportions, and magnitudes of ME | **Loop:**   - Realistic simulation conditions - ME-correction methods are easy to implement | **Loop:**   - Assumed that error-prone follow-up measurements were independent of participant characteristics | **Loop:**   - Use multiple imputation with predictive mean matching when calibrating devices across visits in longitudinal studies |
|  | Self-reported exposure with biomarker validation data?  *Use biomarker informed ME correction* | •**Siddique** 2019 [65]  (methodological/conceptual) | **Siddique:**   - Combined intervention data with auxiliary data from an external biomarker validation study - Used both self-reported dietary intake and recovery biomarkers | **Siddique:**   - ME-corrected estimates were similar to estimates based on urinary sodium measurements in the PREMIER study - This supports the ability of the method to reduce ME | **Siddique:**   - Recovery biomarkers are unavailable for many outcomes relevant to dietary intervention studies - Limited to two time points | **Siddique:**   - Adapt approaches from the longitudinal missing-data literature to ME correction - Develop methods to reduce the number of nonidentified parameters |
|  | Mismeasured covariates include interactions?  *Use interaction-aware regression calibration/ Instrumental Variable method* | •**Strand** 2014 [70]  (methodological/conceptual) | **Strand:**   - Extended regression calibration with instrumental variables (RCIV) to correct ME in covariates and their interaction - Uses unbiased internal surrogates, such as biomarkers, as instrumental variables - Applicable to longitudinal mixed models with repeated outcomes - Derived two estimators: closed-form RCIV1 and computational predicted-value RCIV2 - Derived variance formulas for both estimators | **Strand:**   - Not reported | **Strand:**   - Limited to models with two continuous predictors and their interaction - Assumes one instrumental variable and one unbiased surrogate for each predictor - Further extensions are needed for other model structures - Nonparametric modelling may provide an alternative | **Strand:**   - Avoid ordinary regression calibration and naïve models when interaction terms are included |
|  | Need general correction for ME in regression models?  *Use correction methods informed by repeat/ancillary data* | •**Chambless** 2002 [31]  (applied example)  •**Chambless** 2003 [30]  (applied example)  •**Yanez** 2002 [80]  (simulation) | **Chambless 2002:**   - Estimated reliability of intima–media thickness (IMT) using repeat scans - Applied multivariate regression calibration within mixed models - Used conditional Stein estimators - Transformed data to remove correlation between baseline IMT error and IMT change - Corrected regression estimates for within-person and measurement-process variability   **Chambless 2003:**   - Distinguished variables measured with and without error - Estimated true values of error-prone variables - Transformed the observed data - Applied standard regression models to the transformed data   **Yanez:**   - Compared naïve and bias-corrected regression models - Applied a method-of-moments correction using ME variance estimated from replicate measurements - Used bootstrap standard errors | **Chambless 2002:**   - Longitudinal mixed-effects modelling with repeated intima–media thickness measurements - Adjusts for ME in baseline measurements - Observed progression consistent with previous studies (6.5–10.1 µm/year) - Most risk-factor associations were in the expected direction - Clinical relevance supported by trial evidence   **Chambless 2003:**   - Reduces bias and improves validity of inference - Applicable to multivariate and longitudinal data - Accommodates correlated errors - Consistent and asymptotically normal - Compatible with standard statistical software   **Yanez:**   - Simple, implementable bias correction - Uses replicate measurements to estimate ME - Demonstrated using real longitudinal data - Bias-corrected approach produced valid results in the examples - Implementation available through the BiasFix SAS macro | **Chambless 2002:**   - Small absolute changes in intima–media thickness over follow-up reduce precision - Greater variability in longitudinal change than in cross-sectional measurements - Measurement precision limited by imaging technology - Some associations may remain difficult to detect despite ME correction   **Chambless 2003:**   - Requires external or ancillary data - May increase variance of estimates - Relies on assumptions about the error structure - Computationally complex - Limited correction in very small samples - Corrects only for measured covariates   **Yanez:**   - Assumes classical additive ME - Assumes the error-prone variable is unbiased for the true value - Assumes measurement errors are uncorrelated with the true baseline value - Assumes predictors are measured without error - Requires replicate or validation data - Correction may differ when predictors are also measured with error | **Chambless 2002:**   - Improve measurement precision through advances in imaging and measurement procedures   **Chambless 2003:**   - Use ME-corrected estimators when covariates are measured with error - Incorporate ancillary data or repeated measurements - Prefer corrected models over naïve ordinary least-squares regression - Account for correlated measurement errors - Ensure adequate sample size - Apply transformed data using standard regression software - Consider ME correction when measuring small changes over time   **Yanez:**   - Estimate ME using replicate measurements, validation data, or instrumental-variable information - Use ME-corrected estimators rather than naïve regression when variables used to model change are measured with error |
| **2: Latent variable and joint modelling approaches** | Intensive longitudinal data with many repeated measures?  *Use latent/state-trait or mixed-effects latent models* | •**Nestler** 2024 [48]  (methodological/conceptual)  •**Castro-Alvarez** 2022 [29]  (simulation) | **Nestler:**   - Extended the mixed-effects location-scale (MELS) model to account for ME in outcome variables - Used a common factor model to represent observed outcomes as latent variables - Modelled the resulting latent variable using the MELS framework   **Castro-Alvarez:**   - Used state-trait structural equation models to separate true variation from ME by modelling states and traits as latent variables - Used multilevel models for intensive longitudinal data - Simulated data under known conditions to assess bias in estimates of states and traits | **Nestler:**   - Accounts for ME and between-person differences - Allows covariates to influence all three types of random effects - Provides precise coefficient estimates - Performs comparably to or better than the Bayesian approach   **Castro-Alvarez:**   - Single-level state-trait structural equation models: separate stable trait and dynamic state components while accounting for ME - Multilevel state-trait structural equation models: accommodate intensive longitudinal data with many repeated measurements - Multilevel models use long-format data, reducing overparameterization - Retain the decomposition of trait and state components while accounting for ME | **Nestler:**   - Further model extensions are needed to address additional research questions   **Castro-Alvarez:**   - Single-level state-trait structural equation models: require wide-format data and may become overparameterized with many measurement occasions - Multilevel state-trait structural equation models: more complex to implement and interpret - Multilevel models may remain computationally demanding for very large datasets | **Nestler:**   - Not reported   **Castro-Alvarez:**   - Recommend the Trait-State-Occasion (TSO) model for intensive longitudinal data |
|  | Need to link longitudinal trajectories with a subsequent outcome?  *Use joint modelling to propagate ME* | •**Sayers** 2017 [63]  (simulation) | **Sayers:**   - Compared joint bivariate mixed-effects and structural equation models with two-stage multilevel and simple change-score methods - Joint models simultaneously estimate growth and outcome while accounting for ME - Two-stage and change-score approaches ignore or approximate ME | **Sayers:**   - Demonstrates advantages of joint modelling for longitudinal exposure-outcome analyses with ME - Uses extensive simulations with empirically derived parameter values - Identifies conditions in which two-stage methods may remain adequate | **Sayers:**   - Requires specialist software - Joint models are technically more difficult to fit | **Sayers:**   - Use joint modelling where feasible to account for ME and obtain unbiased estimates of unconditional and conditional effects - Use two-stage multilevel modelling when technical or computational constraints prevent full joint modelling |
|  | Have multiple markers or latent constructs?  *Use SEM or multivariate joint models* | •**Andrinopoulou** 2020 [22]  (methodological/conceptual)  •**Foverskov** 2017 [40]  (applied example) | **Andrinopoulou:**   - Addressed irregular sampling and delayed registry entry - Accounted for ME in longitudinal data and censoring of event data - Considered baseline covariates, confounding, and missing data - Used multivariate joint models - Used cross-validation to assess predictive bias - Assessed robustness to sample size and model specification   **Foverskov:**   - Used structural equation modelling with latent constructs for childhood cognitive ability to account for ME - Used full-information maximum likelihood to reduce bias from missing data - Conducted sensitivity analyses using alternative estimation methods, including maximum likelihood with listwise deletion and robust standard errors | **Andrinopoulou:**   - Novel focus on prediction - Uses joint modelling - Demonstrates robust predictive performance - Adjusts for potential confounders   **Foverskov:**   - Longitudinal data with multiple cognitive assessments from childhood to midlife - Register-based socioeconomic position measures reduce recall bias - Latent-variable modelling accounts for ME | **Andrinopoulou:**   - Focused on time to first event - Prior events may be missing - Relies on assumptions about biomarker structure - Assumes data are missing at random - Computationally demanding - Nutritional markers provided limited improvement   **Foverskov:**   - Findings should not be interpreted causally - Potential for unmeasured confounding, such as genetics and chronic disease - ME in socioeconomic position indicators was not explicitly addressed | **Andrinopoulou:**   - Use the univariate joint model for accurate and precise prediction across different ages and prediction windows   **Foverskov:**   - Include early-life cognitive ability when studying social determinants of later-life cognition - When early cognitive measures are unavailable, use quantitative bias analysis to account for the stability of cognitive differences |
| **3: Likelihood-based and flexible ME models** | Does ME vary across observations?  *Explicitly model heteroscedastic ME rather than assuming constant variance* | •**Nawarathna** 2015 [57]  (simulation)  •**Sadatsafavi** 2008 [61]  (applied example) | **Nawarathna:**   - Developed a heteroscedastic ME model allowing nonlinear relationships between true values - Allows error variance to vary with measurement magnitude - Used Laplace approximation, Gauss–Hermite quadrature, and model approximation via linearization - Evaluated performance through simulations and application to cholesterol data   **Sadatsafavi:**   - Used heteroscedastic regression to account for ME - Modelled residual variance as a function of predictors affecting measurement precision | **Nawarathna:**   - Allows linear or nonlinear recalibration between measurement methods - Accommodates skewed and heavy-tailed data   **Sadatsafavi:**   - Models the mean and variance jointly - Identifies sources of ME - Separates biological from technical variation - Incorporates patient- and setting-specific characteristics into decision-making | **Nawarathna:**   - Assumes equal variance of interaction effects across measurement methods, despite potentially different scales - Without the equal-variance assumption, the linear calibration model is not identifiable   **Sadatsafavi:**   - Not reported | **Nawarathna:**   - Account for heteroscedastic ME and nonlinear relationships between the true values of measurement methods   **Sadatsafavi:**   - Use heteroscedastic regression to analyse longitudinal bone mineral density data - Consider these models for individualized predictions of bone mineral density change and ME |
|  | Do you have multiple sources of data problems simultaneously? (outliers, missings, ME)  *Use robust estimating approaches that jointly address multiple sources of bias* | •**Qin** 2016 [59]  (simulation) | **Qin:**   - Developed a robust estimating-equation approach - Simultaneously addresses outliers, ME, and missing data | **Qin:**   - Requires fewer assumptions about ME - Does not require specification of the distribution of covariates or ME - Does not require construction of correction terms - Easy to implement | **Qin:**   - Not reported | **Qin:**   - Apply the proposed method and extend it to high-dimensional data |
|  | Need flexible estimation of longitudinal trajectories?  *Use flexible smoothing or functional mixed-effects approaches to estimate trajectories while accounting for ME* | •**Andrew** 2007 [21]  (simulation)  •**Chen** 2011 [33]  (methodological/conceptual) | **Andrew:**   - Assessed raw maximum flow-mediated brachial artery measurements, which may overestimate values because of ME - Used kernel-based local linear regression to reduce bias through smoothing - Compared fixed smoothing parameters with automated smoothing using generalized cross-validation - Used simulations to assess bias under different ME and smoothing conditions - Applied smoothing to real-world data to assess bias reduction   **Chen:**   - Decomposed outcome variability into between- and within-subject components using nonparametric and semiparametric covariance estimation - Used likelihood-based selection of smoothing parameters to optimize curve estimation - Used P-splines for population means and varying coefficients to reduce model-misspecification bias | **Andrew:**   - Reduces positive bias from selecting raw maximum diameter values - Flexible smoothing allows assessment of ME effects - Simulations demonstrate bias reduction with appropriate smoothing - Can adjust smoothing for heart rate and ME   **Chen:**   - Flexible estimation of population and individual curves - Between-subject covariance and within-subject variance estimates satisfy positive semidefinite constraints - Modelling the covariance structure improves estimation efficiency - Simulations demonstrate good performance of P-spline estimators - P-spline estimators are asymptotically normal and can achieve efficiency comparable to other smoothing methods with an appropriate number of knots | **Andrew:**   - Optimal smoothing depends on the unknown true maximum, limiting generalizability - Inter-individual variation was not fully addressed in simulations - No general formula for minimum-bias smoothing in real-world data - Smoothing parameters may lead to over- or under-smoothing depending on heart rate, measurement spacing, and ME   **Chen:**   - Asymptotic approximations require large sample sizes - Computationally intensive for large datasets with many repeated measurements - Covariance functions may be difficult for practitioners to interpret - Assumes a common covariance function across subjects unless interaction terms are included | **Andrew:**   - Use nonparametric regression with smoothing for more accurate estimation of flow-mediated dilation in the presence of ME - Carefully select the smoothing parameter to avoid over- or under-smoothing - In research settings, assess associations across a range of smoothing parameters - In clinical settings, use caution when selecting smoothing levels because individual ME may be unknown   **Chen:**   - Use P-spline functional mixed-effects models for flexible modelling of longitudinal trajectories - Decompose between- and within-subject variability to improve efficiency and interpretation - Include covariate interactions when heterogeneity in covariance is expected - Use simulations to select the number of knots and smoothing parameters |
|  | Need baseline outcome error correction in longitudinal models?  *Correct for ME in baseline values of outcome when modelling longitudinal change* | •**Braun** 2014 [25]  (methodological/conceptual)  • **Harrison** 2009 [44]  (methodological/conceptual) | **Braun:**   - Corrected ME in baseline values using conditional modelling to reduce RTM bias - Allowed inclusion of time-constant and time-dependent covariates - Required only selected interactions with time rather than all possible interactions - Used model selection based on the Bayesian information criterion - Applied correction to bivariate models to reduce bias when correlated outcomes are analysed separately - Allowed prediction for new individuals   **Harrison:**   - Fitted a longitudinal mixed model to all repeated CD4 measurements - Applied post-estimation adjustment to account for baseline confounding - Compared the approach with a standard change model including baseline as a covariate - Evaluated performance in a simulation study with 10,000 replicates | **Braun:**   - Improves handling of error-prone baseline values - Flexible and interpretable modelling framework - Bayesian information criterion supports model selection in linear mixed-effects models - Improves robustness of model comparisons   **Harrison:**   - Accounts for baseline confounding - Reduces bias compared with simple change models - Allows valid inference conditional on the underlying baseline value - Applicable to complex longitudinal data - Supports predicted trajectories and informative plots | **Braun:**   - Computationally and methodologically complex - Accounts for baseline ME but not fully for time-varying ME in covariates - Limited generalizability to highly nonlinear trajectories and non-Gaussian outcomes - May be difficult to extend to multivariate settings with more than two outcomes   **Harrison:**   - Requires correct specification of the covariance structure - May be complex to implement - Relies on assumptions about random effects and correlation structures - Less straightforward with complex or non-standard correlation structures - Assumes the sample is representative of the target population | **Braun:**   - Condition on true baseline values - Include only necessary time interactions and relevant covariates - Avoid overly complex time functions to support unbiased and interpretable comparisons of longitudinal change   **Harrison:**   - Use post-estimation adjustment when baseline values are correlated with subsequent change - Prefer this approach over simple change or unadjusted longitudinal models when conditional inference is required |
| **4: Reliability and regression-based change detection and decision frameworks** | Need to distinguish true change from measurement variability?  *Use reliability or regression based change estimators that account for ME and define thresholds for meaningful change* | •**Kennedy** 2020 [78]  (applied example)  •**Andrae** 2023 [20]  (simulation)  •**Dugravot** 2009 [38]  (applied example)  •**Jackson** 2004 [47]  (applied example) | **Kennedy:**   - Used structural equation modelling to distinguish true change from ME   **Andrae:**   - Raw change score: no ME adjustment; susceptible to RTM - Lord and Novick regression estimator: uses pre- and post-test reliability to account for ME and RTM - Cronbach and Furby complete estimator: extends the Lord and Novick estimator using additional covariates or high-quality anchor items - True score: used as an unbiased benchmark - Used simulations to compare corrected estimators with raw change and true scores - Defined significant change using a 95% confidence interval based on the standard error of estimation   **Dugravot:**   - Used errors-in-variables regression with test–retest reliability to correct baseline-adjusted estimates for ME - Conducted sensitivity analyses assuming higher ME   **Jackson:**   - Used general linear models to model change in treadmill performance | **Kennedy:**   - Uses reliable test measures to distinguish meaningful change from ME - A ±2 standard-error threshold accounts for ME when identifying meaningful effects   **Andrae:**   - Incorporates ME using reliability estimates from both measurement occasions and their difference - Cronbach and Furby estimator can incorporate additional covariates - Regression estimators reduce dispersion compared with raw change scores - Lord and Novick estimator showed the smallest deviation from true scores and the greatest precision - Standard-error-based limits closely matched individual standard errors from computerized adaptive testing - Provides a practical alternative to raw change scores for detecting individual change   **Dugravot:**   - Applies correction for ME - Compares unadjusted and baseline-adjusted methods   **Jackson:**   - Explicitly distinguishes true change from ME | **Kennedy:**   - Conditioned pain modulation showed only fair reliability, with substantial within-subject variability - Sham conditional stimulus showed poor reliability and may reflect distraction or expectation effects   **Andrae:**   - Cronbach and Furby estimator showed moderate to large deviations for some individual scores - Z-score-transformed sum scores showed larger deviations than the Lord and Novick estimator - Methods do not fully account for item-level variability across latent traits   **Dugravot:**   - Two-wave design limits modelling of nonlinear trajectories - Assumes normally distributed ME   **Jackson:**   - Fitness was measured indirectly using treadmill time | **Kennedy:**   - Use a ±2 standard-error-of-measurement threshold to classify conditioned pain modulation response - Report individual-level responses in addition to group averages - Use repeated baseline test-stimulus measurements to estimate the standard error of measurement for each study population - This approach may reduce misclassification due to ME   **Andrae:**   - Consider regression-based change estimates to improve measurement sensitivity   **Dugravot:**   - Interpret baseline-adjusted analyses cautiously because of potential RTM - Use ME-correction methods when baseline variables are measured with error - Collect multiple waves to better model cognitive trajectories   **Jackson:**   - Use multivariate longitudinal modelling when analysing serial fitness measurements - Distinguish true behavioural and physiological change from ME |
|  | Need decision rules that account for consequences of misclassification of change?  *Use Bayesian/decision analytic criteria* | •**Sadatsafavi** 2008 [62]  (methodological/conceptual) | **Sadatsafavi:**   - Developed an optimal decision criterion using Bayesian and decision-theoretic principles - Maximizes a utility function that rewards correct and penalizes incorrect classification of change | **Sadatsafavi:**   - Incorporates prior knowledge about likely true change - Can be tailored according to the relative value assigned to correct and incorrect classifications | **Sadatsafavi:**   - Normality assumption may not hold when the prior distribution is estimated from real-world data - Assigns the same score to all individuals within a given classification quadrant - Assumes ME is accurately known, although in practice it is often estimated from limited in vivo precision data | **Sadatsafavi:**   - Use Bayesian and decision-theoretic approaches to support more rigorous interpretation of change in monitoring practice |
|  | Need to define or evaluate minimally important change (MID)?  *Use anchor-based MID methods cautiously and assess baseline severity effects* | •**Browne** 2010 [27]  (applied example) | **Browne:**   - Developed a cohort and measurement model - Applied the Transition Rating Index (TRI) model - Applied a validity model - Applied a baseline-severity association model based on Oldham’s method | **Browne:**   - Anchor-based method: supports estimation of minimal change and generation of minimal important difference values - Oldham’s method: examines the influence of baseline severity on minimal important difference estimates - Uses correlation analysis and variance-ratio tests to assess baseline-change relationships - Demonstrated robustness and supports estimation of a single minimal important difference value | **Browne:**   - TRI method showed low validity for some measures - Moderate construct validity - Weak association with change scores | **Browne:**   - Use Oldham’s method to evaluate minimal important difference values and the effect of baseline severity - Interpret TRI derived values cautiously, particularly for measures with weak construct validity |
| **5: Bias evaluation, design guidance, and methodological critique** | Need guidance on handling baseline values when analysing change?  *Consider the role of baseline values before adjustment; choose models according to the inferential goal* | •**McArdle** 2009  (simulation) [56]  •**Blance** 2007 [24]  (applied example)  •**Edland** 2000 [39]  (methodological/conceptual)  •**Grijalva** 2015 [42]  (simulation)  •**Wampold** 2007 [75]  (simulation)  •**Jones** 2016 [48]  (applied example)  •**Chiolero** 2013 [79]  (simulation) | **McArdle:**   - Compared blood-pressure response models with and without baseline blood pressure as a predictor - Assessed resulting bias from baseline adjustment   **Blance:**   - Used multilevel modelling to correct bias   **Edland:**   - Modified Blomqvist’s estimator using a reference age rather than time since baseline - Used method-of-moments and maximum likelihood estimation for unbalanced data - Used centred designs to reduce covariance between slope and intercept errors - Distinguished descriptive/predictive from etiological inference   **Grijalva:**   - Compared change-score analysis with linear and nonlinear analysis of covariance - Examined models with and without robust variance estimation - Simulated baseline matching to assess bias, coverage probability, and standard errors   **Wampold:**   - Used simulations based on the study design of the NIMHTDCRP dataset - Assessed power to detect residual patient intercept variance when baseline was used to predict patient intercepts - Examined the effect of anchoring on estimates of therapist effects   **Jones:**   - Used correlation and linear regression to assess baseline–response relationships - Compared baseline with pre-baseline glycated haemoglobin measurements taken 1–26 weeks earlier - Examined pre-baseline to baseline change to estimate RTM - Applied baseline adjustment and a bias-correction method using repeated baseline measurements to account for ME   **Chiolero:**   - Compared multiple methods under varying levels of ME and between-individual variability in change - Methods included crude correlation, variance comparison, Oldham’s method, repeated-measurement methods, Blomqvist’s method, and linear random-effects regression | **McArdle:**   - Explicitly considers underlying causal mechanisms   **Blance:**   - Adjusts for baseline values - Flexible multilevel modelling framework - Corrects for some baseline-related effects - Simulations demonstrate adequate statistical power   **Edland:**   - Extends Blomqvist’s method to unbalanced longitudinal data - Clarifies when slope-on-level analysis is appropriate - Distinguishes predictive/descriptive analyses from etiological inference   **Grijalva:**   - Nonlinear analysis of covariance with robust variance estimation is resistant to baseline imbalance, nonlinear associations, and small ME - Provides accurate effect estimates - Baseline matching reduces bias and sensitivity to assumption violations - Matching improves robustness of other analytical approaches   **Wampold:**   - Simulation with known parameter values allows direct assessment of bias introduced by anchoring   **Jones:**   - Large prospective observational cohorts - Findings assessed across two independent cohorts and two therapies - Repeated pre-baseline glycated haemoglobin measurements allowed assessment of ME and RTM - Bias correction supported the appropriateness of baseline adjustment   **Chiolero:**   - Directly compares several established methods - Examines multiple simulation scenarios with realistic BMI growth parameters - Explicitly examines ME and variability in change - Provides guidance according to the number of measurement waves and available ME information | **McArdle:**   - Not reported   **Blance:**   - Baseline adjustment in nonrandomized studies may be affected by Lord’s paradox - Limited by small sample sizes and assumptions of multilevel models - May be difficult to interpret and implement   **Edland:**   - Original Blomqvist method may be biased with pooled age cohorts or unbalanced data - Two-wave designs cannot estimate variance components required for the full Blomqvist method - Ceiling and floor effects may limit interpretation   **Grijalva:**   - Change-score analysis may be biased without matching and overly conservative after matching - Linear analysis of covariance may be biased with baseline imbalance - Nonlinear analysis of covariance without robust variance estimation has poorer confidence-interval coverage and is sensitive to ME - Performance of all methods deteriorates with moderate ME   **Wampold:**   - Not reported   **Jones:**   - Clinical treatment decisions may exaggerate RTM   **Chiolero:**   - Assumed linear growth and no confounding - Relatively small simulation sample (n = 500) - Simulated distributions may not represent all real-world settings - Linear random-effects models require multiple measurements - No satisfactory method identified for two-wave data when linear growth is inappropriate | **McArdle:**   - Consider underlying causal mechanisms when selecting the analytical strategy - Use approaches that support unbiased replication across genetic association studies   **Blance:**   - Use multilevel modelling for repeated-measures studies, particularly when baseline effects or hierarchical data structures are present   **Edland:**   - Use Blomqvist’s method for predictive or descriptive purposes rather than aetiological inference in chronic disease - For two-wave designs, use Oldham-style centred analysis to test for slope–level relationships   **Grijalva:**   - For continuous outcomes with potential baseline differences, consider matching followed by nonlinear analysis of covariance with robust variance estimation   **Wampold:**   - Avoid assuming that the initial longitudinal observation is measured without error - Recognize that tests for zero error in the initial observation may be underpowered - Avoid anchoring the initial observation when it is measured with error, as this may bias estimates   **Jones:**   - Adjust for baseline glycated haemoglobin when analysing predictors of treatment response - Standard baseline regression adjustment is appropriate when repeated baseline measurements are unavailable - Bias correction using repeated baseline measurements supports baseline adjustment - Expressing change as a percentage of baseline does not sufficiently account for baseline confounding   **Chiolero:**   - Use multiple measurements whenever possible - With >2 waves, use linear random-effects modelling - With two waves and accurate ME information, Blomqvist’s method provides accurate estimates - Interpret baseline-change associations cautiously, considering ME and variability in change |
|  | Need guidance for modelling and interpreting complex longitudinal measurements?  *Choose modelling approaches that account for repeated measurements, measurement variability, and other measurement challenges* | •**Howe** 2016 [46]  (applied example)  •**Weuve** 2015 [76]  (methodological/conceptual) | **Howe:**   - Used multilevel models with random intercepts and random slopes - Modelled trajectories using linear splines   **Weuve:**   - Used multivariate latent-variable or ME models for multiple measurement problems - Used instrumental-variable analyses - Constructed composite scores from multiple neuropsychological assessments - Evaluated differential item functioning and applied statistical corrections when needed | **Howe:**   - Accounts for correlated repeated measurements and individual heterogeneity - Accommodates irregular measurement timing and unequal numbers of observations - Includes participants with incomplete data under a missing-at-random assumption - Provides interpretable, period-specific growth rates - Reduces dimensionality and collinearity of growth data - Can explicitly model age-dependent ME   **Weuve:**   - Not reported | **Howe:**   - Assumes piecewise linear growth, which may be biologically implausible - Results depend on subjective choice and placement of knot points - Limited ability to capture short-term growth spurts - Does not naturally account for residual autocorrelation with irregular measurement intervals - Complex variance–covariance structures may cause convergence problems, particularly with sparse measurements   **Weuve:**   - Limited empirical and theoretical evidence comparing the performance of many proposed approaches in dementia research - Statistical approaches for handling practice effects remain controversial | **Howe:**   - Use linear-spline multilevel modelling for longitudinal growth data with irregular measurements - Consider nonlinear models when biological milestones are the primary outcome   **Weuve:**   - Follow the MELODEM (MEthods in LOngitudinal research on DEMentia) guidelines to acknowledge measurement-related challenges and justify analytical decisions |
|  | Concerned that observed transitions or change classifications may be artefacts of ME?  *Use repeated measurements and interpret apparent transitions cautiously, especially in two-wave studies where ME may create spurious change* | •**Houlle** 2007 [45]  (simulation)  •**Edland** 2000 [39]  (methodological/conceptual) | **Houlle:**   - Simulated true headache scores for 2,000 individuals - Added normally distributed ME to generate observed scores at two time points - Assessed apparent chronification and remission arising solely from ME and regression effects - Evaluated three true-score distributions, including smoothed negative binomial distributions with and without rounding   **Edland:**   - Modified Blomqvist’s estimator using a reference age rather than time since baseline - Used method-of-moments and maximum likelihood estimation for unbalanced data - Used centred designs to reduce covariance between slope and intercept errors - Distinguished descriptive/predictive from etiological inference | **Houlle:**   - Systematically examines different levels of ME - Uses realistic headache-frequency distributions from population data - Demonstrates the risks of interpreting chronification or remission from only two time points   **Edland:**   - Extends Blomqvist’s method to unbalanced longitudinal data - Clarifies when slope-on-level analysis is appropriate - Distinguishes predictive/descriptive analyses from etiological inference | **Houlle:**   - Assumes constant ME across headache frequency, which may not reflect real-world measurement - Two time points cannot fully capture longitudinal headache trajectories   **Edland:**   - Original Blomqvist method may be biased with pooled age cohorts or unbalanced data - Two-wave designs cannot estimate variance components required for the full Blomqvist method - Ceiling and floor effects may limit interpretation - Etiological inference may not be justified for chronic progressive conditions | **Houlle:**   - Use repeated, reliable measurements, such as daily headache diaries, to reduce ME - Use multiple time points rather than two measurements to better capture true trajectories   **Edland:**   - Use Blomqvist’s method for predictive or descriptive purposes rather than aetiological inference in chronic disease - For two-wave designs, use Oldham-style centred analysis to test for slope–level relationships |

^a^Reference numbers for all publications refer to the main text references

^b^List of acronyms: BMI = body mass index; CD4 = CD4 cell count; IMT = intima–media thickness; ME = measurement error; MELODEM = MEthods in LOngitudinal research on DEMentia; MELS = mixed-effects location-scale; RCIV = regression calibration with instrumental variables; RTM = regression to the mean; SAS = Statistical Analysis System; TRI = Transition Rating Index; TSO = Trait–State–Occasion.

| **Applied studies outcome** | **Studies that adjusted for baseline outcome value** | **Studies that did not adjust for baseline outcome** | **Studies reporting both approaches** |
| --- | --- | --- | --- |
| BMI^a^: N=55 | 23 (42%) | 27 (49%) | 5 (9%) |
| Weight: N=29 | 20 (69%) | 8 (28%) | 1 (3%) |
| Blood Pressure: N=22 | 10 (45%) | 12 (55%) | 0 |
| Waist Circumference: N=13 | 8 (61%) | 4 (31%) | 1 (8%) |
| HDL-C: N=14 | 4 (29%) | 9 (64%) | 1 (7%) |
| Triglycerides: N=10 | 3 (30%) | 7 (70%) | 0 |
| Metabolic risk factor score: N=1 | 1 (100%) | 0 | 0 |
| HbA1c: N=5 | 0 | 5 (100%) | 0 |

**Table S3**: Use of baseline outcome adjustment according to outcome type in Applied studies.

^a^BMI, body mass index; HDL-C, high-density lipoprotein cholesterol; HbA1c, glycated haemoglobin.
